# A Bayesian Partition Method for Detecting Pleiotropic and Epistatic eQTL Modules

**DOI:** 10.1371/journal.pcbi.1000642

**Published:** 2010-01-15

**Authors:** Wei Zhang, Jun Zhu, Eric E. Schadt, Jun S. Liu

**Affiliations:** 1 UBS Equities, Stamford, Connecticut, United States of America; 2 Rosetta Inpharmatics, LLC, Merck & Co., Inc., Seattle, Washington, United States of America; 3 Sage Bionetworks, Seattle, Washington, United States of America; 4 Pacific Biosciences, Menlo Park, California, United States of America; 5 Department of Statistics, Harvard University, Cambridge, Massachusetts, United States of America; Washington University School of Medicine, United States of America

## Abstract

Studies of the relationship between DNA variation and gene expression variation, often referred to as “expression quantitative trait loci (eQTL) mapping”, have been conducted in many species and resulted in many significant findings. Because of the large number of genes and genetic markers in such analyses, it is extremely challenging to discover how a small number of eQTLs interact with each other to affect mRNA expression levels for a set of co-regulated genes. We present a Bayesian method to facilitate the task, in which co-expressed genes mapped to a common set of markers are treated as a module characterized by latent indicator variables. A Markov chain Monte Carlo algorithm is designed to search simultaneously for the module genes and their linked markers. We show by simulations that this method is more powerful for detecting true eQTLs and their target genes than traditional QTL mapping methods. We applied the procedure to a data set consisting of gene expression and genotypes for 112 segregants of *S. cerevisiae*. Our method identified modules containing genes mapped to previously reported eQTL hot spots, and dissected these large eQTL hot spots into several modules corresponding to possibly different biological functions or primary and secondary responses to regulatory perturbations. In addition, we identified nine modules associated with pairs of eQTLs, of which two have been previously reported. We demonstrated that one of the novel modules containing many daughter-cell expressed genes is regulated by *AMN1* and *BPH1*. In conclusion, the Bayesian partition method which simultaneously considers all traits and all markers is more powerful for detecting both pleiotropic and epistatic effects based on both simulated and empirical data.

## Author Summary

Genome-wide association studies (GWAS) have yielded several causal genes for many human diseases. However, the mechanisms underlying how DNA variations affect disease phenotypes have not been well understood in many cases. Gene expression is intermediate between DNA and clinical endpoints. Linking DNA variation and gene expression variation, often referred to as “expression quantitative trait loci (eQTL) mapping”, has yielded clues of mechanisms and pathways by which DNA variations impact phenotypes. Because of the large number of genes and genetic markers in such analyses, it is extremely challenging to discover how a small number of eQTLs interact with each other to affect mRNA expression levels for a set of co-regulated genes. We present a Bayesian method to identify genetic interactions and more eQTLs by treating co-expressed genes as a module. Our method provides a tool to study genetic interactions in human disease models.

## Introduction

Studies in the genetics of gene expression combine gene expression and genotype data in segregating populations to detect loci linked to variations in RNA levels. These loci are referred to as expression quantitative trait loci (eQTL). To date, eQTL studies have been pursued in a number of species ranging from yeast to mouse and human [1]–[3]. A common theme of these studies is to treat thousands of gene expression values as quantitative traits and conduct QTL mapping for all of them.

Most eQTL studies are based on linear regression models [4] in which each trait variable is regressed against each marker variable. The p-value of the regression slope is reported as a measure of significance for the association. In the context of multiple traits and markers, procedures such as false discovery rate (FDR) controls [5] can be used to quantify family-wise error rates. Despite the success of this type of regression approach, a number of challenging problems remain. First, these methods can not easily assess the joint effect of multiple markers beyond additive effects. Storey *et al.* [5] developed a step-wise regression method to find eQTL pairs, then Zou and Zeng improved it [6]. This procedure, however, tends to miss eQTL pairs with small marginal effects but a strong interaction effect. There are methods for detecting eptistatic effects without main marginal effects [7]–[8]. However, their applications are limited to a few clinical traits instead of thousands of expression traits due to computational constraints. Second, there are often strong correlations among expression levels for certain groups of genes, partially reflecting co-regulation of genes in biological pathways that may respond to common genetic loci and environmental perturbations [2], [9]–[11]. Previous findings of eQTL “hot spots”, i.e., loci affecting a larger number of expression traits than expected by chance, and their biological implications further enhance this notion and highlight the biological importance of finding such gene “modules”. Mapping genetic loci for multiple traits simultaneously is more powerful than mapping single traits at a time [12]. Although for a known small set of correlated traits, one can conduct QTL mapping for the principal components [13], this method becomes ineffective when the set size is moderately large or one has to enumerate all possible subsets. An alternative approach is to identify subsets of genes by a clustering method, and then fit mixture models to clusters of genes [14]. The eQTL mapping then depends on whether the distance metric used by the clustering method is appropriate, whether the method can find the right number of clusters.

We address these issues by modeling the joint distribution of all genes and all markers simultaneously. Under a Bayesian framework, we introduce three sets of latent indicator variables for genes, markers, and individuals, and then systematically infer the association between groups of genes and sets of markers. In this framework, correlated expression traits and their associated set of markers are treated as a module so as to account for epistatic interactions and pleiotropic effects. Parameters of interest are the partitions of genes and markers into modules, and the partition of individuals into different types that correspond to the relationships between expression levels and marker genotypes in a given module. A Markov chain Monte Carlo (MCMC) algorithm is designed to traverse the space of all possible partitions. Simulation studies show that the proposed method achieves significantly improved power in detecting eQTLs compared to traditional regression-based methods. A particular strength of our method is its ability to detect epistasis with high power when the marginal effects are weak, addressing a key weakness of all other eQTL mapping methods.

We applied our method to a previously described data set consisting of gene expression and genotypes data for 112 segregants from a cross between laboratory (BY) and wild (RM) strains of *S. cerevisiae* [15]. In addition to identifying several modules linked to single eQTLs that are consistent with previous reports [1],[11],[16], our method dissected large eQTL hot spots into different modules that correspond to different causal regulators or to primary and secondary responses to causal regulators. In addition, we detected nine modules under the control of two genetic loci. One of these modules corresponds to a previously verified result regarding the interaction between *GPA1* and *MAT* [5],[16]. another is regulated by both *ZAP1* expression and genotype, consistent with previously described results [17]. The other seven modules represent novel findings. Three of these appear to be artifacts of cross-hybridization in microarray experiments; while another exhibits strong epistatic interactions between two loci consisting of many daughter-cell expressed genes that we predict are under the regulation of *AMN1* and *BPH1*.

## Results

### Overview of Bayesian partition method

**Figure 1:**
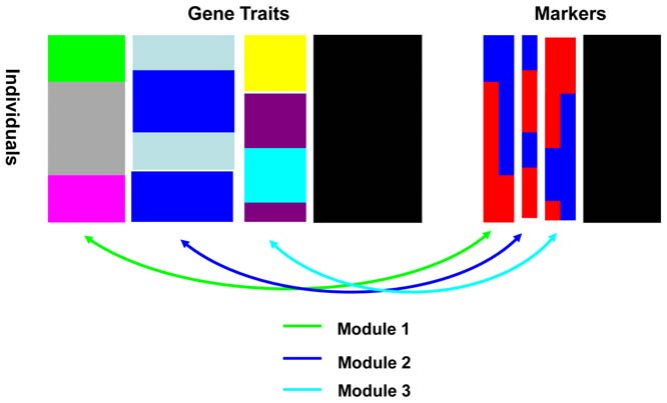
An illustration of the Bayesian partition model. Each row represents an individual and the columns represent gene expression traits (left) and markers (right). Data is partitioned into three modules plus a null module. Module 1 has two markers associated with a group of genes, represented by a link in green color. In this module individuals are partitioned into three individual types. Genes in module 2 are associated with one marker, represented by a link in blue color. Individuals in module 2 are partitioned into two individual types. Similarly module 3 has two markers linked with a group of genes, represented by a link in red color. Individuals in module 3 are partitioned into three individual types. Genes and markers in the null module are drawn in black. Note that different modules have different individual partitions.

**Figure 2:**
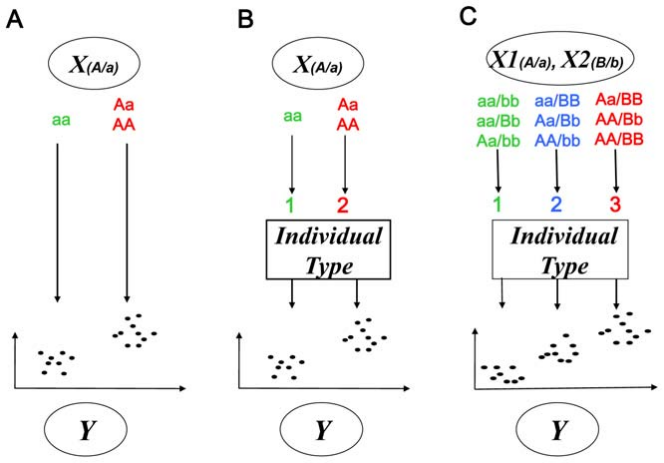
Comparison of different models for associating genotypes and phenotypes. (A) the regression model; (B) the Bayesian partition (BP) model with a single biallelic marker ; (C) the BP model with two interactive biallelic markers. In the regression model, gene expression values (Y) are regressed onto marker genotypes (X). If the marker has a dominant effect on the gene expression, the regression implicitly partitions the expression values into low and high groups corresponding to genotypes aa for, say, low expression and Aa or AA for high expression. In the BP model, a latent variable, denoted here as “Individual Type”, is introduced and conditional on this variable the gene expression traits and marker genotypes are independent. In the case of a single marker, two individual types exist, colored here as green and red. In (c), gene expression is linked with a set of two biallelic markers. In this instance, individuals are partitioned into three types, colored here as green, blue and red, corresponding to low, medium, and high expression levels, respectively.

We define a ***module*** as a set of gene expression traits (referred to as “genes” henceforth) and a set of genetic markers (e.g., SNPs) such that the variation of the gene expression traits is associated with the variation of the markers, as shown in Figure 1. This association between multiple genes and markers is characterized by a latent indicator variable, individual type, conditional on which the trait and marker variables are independent of each other. The individual type latent variable can be viewed as representing a certain combination of markers that induces changes in expressions of a certain set of genes across different individual types. In the simplest case with a single marker, the individual type could correspond to a dominant genetic model, as illustrated in Figure 2A. In this instance, our model is mathematically equivalent to the regression model (Figure 2B). In the case of two markers associated with gene expression traits, there could be two to nine individual types (various genotype combinations). Figure 2C illustrates a case with three individual types: 1) high expression values associated with red-colored genotype combinations, 2) medium expression values with blue-colored combinations, and 3) low expression values with green-colored combinations. The goal of the Bayesian partition method is to simultaneously partition genes and SNPs into modules. The details of the Bayesian partition model are described in the Methods section.

### Simulation studies

**Figure 3:**
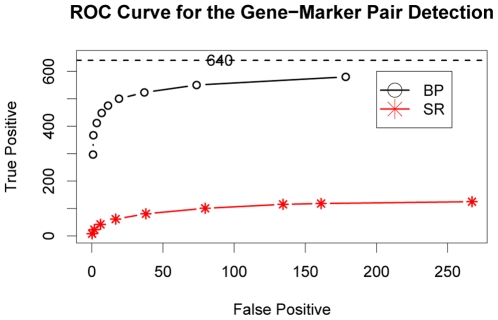
Comparison of the receiver operator characteristic (ROC) curves for the gene-marker pair detection obtained by our Bayesian partition method (BP) and the two-stage regression method (SR). Different points along the ROC curves represent the false positive and true positive counts averaged over 100 simulations at different posterior probability thresholds (for BP) or at different FDR thresholds (for SR). There are 40 genes in each of the eight modules which are linked to two markers and thus the total number of the true positive gene-marker pairs is 640.

To test the effectiveness of our method, we simulated 120 individuals with 500 binary markers and 1000 expression traits in the context of inbred cross of haploid strains. There are eight modules (summarized in Table 1), each consisting of 40 genes, simulated from different epistasis models based on the linear regression framework, which is different from the posited Bayesian model in our analysis. The genotypic means and frequencies for the two loci used in the simulation are listed in Table 2. We repeated the simulation 100 times and analyzed the simulated data using two methods: (1) our Bayesian partition method using parallel tempering [18] with 15 temperature ladders, referred to as BP; (2) the two-stage regression method of Storey *et al* [5], referred to as SR. Details of the simulation and implementation of these two methods are described in the *Supplemental Material*. As shown from the receiver operating characteristic (ROC) curves in Figure 3, BP achieved a significantly higher power to detect eQTLs compared to SR. For example, allowing for 50 false positives, BP detected more than 500 (out of 640) true gene-marker pairs, whereas SR only detected ∼100 true pairs and became plateaued even with many more false positives allowed. There are likely two reasons for this. First, we modeled the co-regulated genes as a module so that information from all genes in a given module could be aggregated to improve the signal. Multiple trait mapping has proven to be more powerful than single trait mapping [12] in the regression framework. Second, we modeled epistatic interactions explicitly so that markers with weak marginal but strong interactive effects could be detected.

**Table 1:** Simulation design and genetic variance decomposition of different models.

Module	Model^a^	% of Var.^b^	Locus 1^c^	Locus 2^d^	Epistasis^e^
A	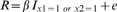	0.153	0.338	0.339	0.333
B	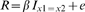	0.158	0.052	0.052	0.895
C	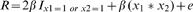	0.160	0.466	0.441	0.088
D	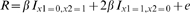	0.161	0.133	0.128	0.739
E	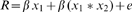	0.132	0.748	0.138	0.128
F	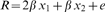	0.169	0.736	0.231	0.043
G	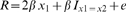	0.168	0.743	0.050	0.211
H	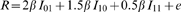	0.168	0.131	0.048	0.821

a The regression model that was used to generate the “core gene” in each module.

b The average percentage of variation of genes in the module explained by the true model.

c The average percentage of genetic variance explained by the first locus.

d The average percentage of genetic variance explained by the second locus.

e The average percentage of genetic variance explained by epistasis. In all modules, the heritability of the “core gene” is 0.6 and the average correlation of the module genes with the “core gene” is 0.5.

**Table 2:** Genotypic means and frequencies for a two-locus model used in the simulation studies.

		Locus 2		Mean
		B	b	
Locus 1	A	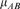	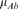	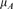
		( 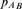 )	( 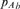 )	
	a	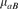	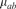	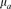
		( 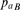 )	( 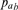 )	
Mean		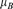	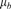	

The contrast of the performances of these two methods is most prominent when the marginal effect is weak. For example, in modules B, D and H, the rate of true positive detections of SR never exceeded 5% even at the generous FDR threshold of 90%. In modules E, F, and G where the major marker explains more than 70% of the genetic variation, SR detected the major marker in nearly 50% of the simulations at the 50% FDR threshold, but not the minor marker. In contrast, BP performed superiorly and robustly in all eight modules. The module by module comparisons are detailed in the *Supplemental Material* Text S1 and shown in Supplementary Figure S1.

**Figure 4:**
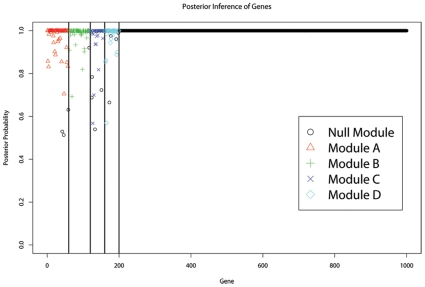
The posterior probability plot. The height of each point is the posterior probability for the most likely classification of the gene; and the shape/color of the point represents the module type of the classification. The first 200 genes are those in one of the four non-null modules, separated by vertical lines.

Figure 4 provides a graphical view of the BP result for another simulated dataset with 120 individuals, 1000 genes, and 500 markers. Four distinct modules, with 60, 60, 40, and 40 genes, and controlled by 3, 2, 1, and 2 markers, respectively (shown in Supplementary Table S1), are simulated similarly as in the previous example (more details in the *Supplemental Material* Text S1). The shape and height of a point represent the most probable module classification and the corresponding maximum posterior probability of a gene. We see that all of the “background” genes were correctly classified according to their highest posterior probabilities. Most genes in the four non-null modules were also correctly classified, other than a very few ones that were classified into the null module, most likely due to their weak signals. BP also correctly identified the truly associated markers of the four modules with high posterior probabilities (shown in Supplementary Table S2).

### Yeast eQTL modules – a re-examination of the landscape of genetic complexity

We applied our Bayesian method to a data set consisting of gene expression and genotypes for 112 segregants from a cross between laboratory (BY) and wild (RM) strains of *S. cerevisiae* [15] and detected 29 modules of genes and their associated markers (Methods). Among these 29 modules, 20 are linked to a single eQTL while the remaining nine are linked to two eQTLs. Three of the nine linking to two eQTLs give rise to significant epistatic interactions between the two loci. Twenty-six of the 29 modules significantly overlap (corrected p-value<0.05) with at least one of the 13 gene groups previously reported as mapping to eQTL hot spots [11]. We also tested each of these modules for enrichment using GO terms, a yeast knockout compendium [19], and transcription factor binding sites [20]. At p-value<0.05 after multiple testing correction, 21 modules have at least one GO term enrichment; 22 modules overlap with at least one knockout signature, and 13 modules are enriched for at least one transcription factor binding site. The result is summarized in Table 3 and a breakdown result is in Supplementary Table S3. In contrast, the LOD score distributions of transcripts at the associated markers under the “single-transcript-single-marker” model are shown in Supplementary Figure S2. Our Bayesian method identifies significantly more weak gene-marker associations than the simple model. These GO enrichments support the biological relevance of different modules detected by our method. Each module is described in detail in the *Supplemental Material* Text S1.

**Table 3:** Summary of the 29 modules that were detected in the yeast data set.

Module	Size^a^	Loci^b^	GO category^c^	KO^d^	TFBS^e^	eQTL hot spot^f^
1	38	Chr II: 548401		0	0	
2	33	Chr II: 548401		1	0	2****
3	16	Chr II: 548401	cell wall (sensu Fungi)**	6	3	2***
		Chr III: 177850				3*
4	137	Chr II: 548401	Nucleolus****	9	0^g^	2****
5	75	Chr II: 548401		0	1	2***
6	38	Chr II: 602012	Protein disulfide isomerase activity**	2	1	8***
7	83	Chr III: 79091	‘de novo’ IMP biosynthesis**	17	2	4 ****
		Chr XV: 170945				10**
						12***
8	69	Chr III: 79091	histidine biosynthesis*	53	2	4****
9	61	Chr III: 79091		7	0	4***
10	18	Chr III: 81832	branched chain family amino acid biosynthesis*	18	1	4****
11	52	Chr III: 81832	nuclear nucleosome***	3	2	
		Chr VIII: 84437				
12	13	Chr III: 201166	Regulation of transcription from RNA polymerase II promoter*	1	0	4****
		Chr VIII: 111679				5*
13	9	Chr III: 201166		10	3	4****
						5**
14	13	Chr V: 116530	‘de novo’ pyrimidine base biosynthesis**	4	0	6****
15	44	Chr VIII: 111690	Mating projection tip***	20	3	7****
16	10	Chr X: 22315	aldehyde metabolism***	0	0	
		Chr VI: 28041				
17	11	Chr XII: 659357		12	0	8**
		Chr XIII: 430164				
18	45	Chr XII: 662627	ergosterol biosynthesis****	6	1	8****
		Chr III: 79091				
19	34	Chr XII: 105609	telomerase-independent telomere maintenance***	11	0	9****
		Chr IV: 1525327				
20	21	Chr XIII: 49903		4	2	10***
		Chr X: 327852				
21	81	Chr XIV: 449639	endoplasmic reticulum***	2	0	1*
22	52	Chr XIV: 486861	structural constituent of ribosome****	2	0	11****
23	68	Chr XIV: 486861	Arp2/3 protein complex**	0	0	11**
24	39	Chr XIV: 449639	nuclear pore organization and biogenesis*	0	0	11****
25	77	Chr XIV: 486861	mitochondrial inner membrane**	0	0	11****
26	83	Chr XV: 170945	response to stress***	33	1	12***
27	45	Chr XV: 170945		0	0	12****
28	74	Chr XV: 170945	Fructose transporter activity*	4	0	12****
29	42	Chr XV: 563943	respiratory chain complex III (sensu Eukaryota)****	10	5	13****

a Number of genes in each module.

b The chromosome positions of markers associated with each module.

c The most significant GO terms. A total of 510 GO terms of sizes 5 to 300 were tested. Multiple testing corrected (Fisher Exact Test p-value
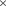
510) p-values less than 0.05 are displayed at four different levels indicated by *. *: 10^−3^∼0.05; **: 10^−5^∼10^−3^; ***: 10^−10^∼10^−5^; ****0∼10^−10^.

d Number of knockout signatures that overlap with each module. 287 knockout signatures [19] were tested and the p-value cut-off is 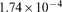 (0.05/287).

e Number of the transcription factors whose binding sites are enriched in each module. 119 transcription factor binding sites [20] were tested and the p-value cut-off is 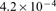 (0.05/119).

f Overlapped eQTL hot spots. Multiple testing corrected (Fisher Exact Test p-value
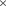
13) p-values at cut-off 0.05 are displayed in four different levels indicated by *.

g Module 4 is enriched with *de novo* motifs PAC and RRPE.

*: 10^−3^∼10^−2^.

**: 10^−5^∼10^−3^.

***: 10^−10^∼10^−5^.

****: 0∼10^−10^.

#### Modules linked to complex eQTL hot spots

Several modules are linked to loci that correspond to previously identified eQTL hot spots [11]. For example, modules 26–28 are linked to a locus on chromosome XV that is coincident with eQTL hot spot 12, with all modules significantly overlapping with genes linked to this locus (p-value = 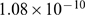, 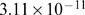, and 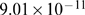, respectively). The average intra-module correlation for module 26 (0.731) is higher than that for modules 27 (0.409) and 28 (0.459). *PHM7* was previously identified and validated as a causal regulator for this hot spot [10]. The *PHM7* knockout signature significantly overlaps with modules 26 and 28 (p-value = 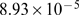 and 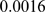, respectively). When compared to a previously constructed yeast knockout compendium [19], module 26 overlaps with 33 knockout signatures, while module 28 overlaps with only four of the knockout signatures (three of the four also overlap with module 26). Application of a causality test procedure [21] revealed that 52 genes (out of 83) in module 26 were supported as causal for at least one gene in module 28, while only six genes (out of 74) in module 28 were supported as causal for at least one gene in module 26 (shown in Supplementary Figure S3). These results indicate that genes in module 26 serve as the primary response to the causal perturbation of *PHM7* and genes in module 28 serve as the secondary response. Other causal regulators for module 27 that are independent of *PHM7* may exist.

#### Modules linked to two loci

Our results provide a number of positive controls that illustrate how our method can dissect complex eQTL hot spots into different modules and detect modules with complex genetic regulation. As summarized above, nine of the 29 modules we identified are linked to two eQTLs. Modules 3, 12 and 16 have significant epistatic interactions (p-value = 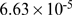, 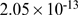 and 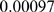, for the interaction terms, respectively) between the two loci. Modules 12 and 20 were previously reported in the literature [16]–[17]. Among the other seven novel modules, three of them (modules 16, 17 and 19) are likely due to cross-hybridization (see details in *Supplemental Material* Text S1). Module 3, which consists of many daughter cell expressed genes and is linked to two eQTLs with a significant epistatic interaction, is predicted to be under the regulation of *AMN1* and *BPH1*, each located near the two eQTL loci. Modules 7 and 18 are each mapped to two previously detected eQTL hot spots suggesting that genes in these two modules are under the control of multiple mega-regulators.

**Figure 5:**
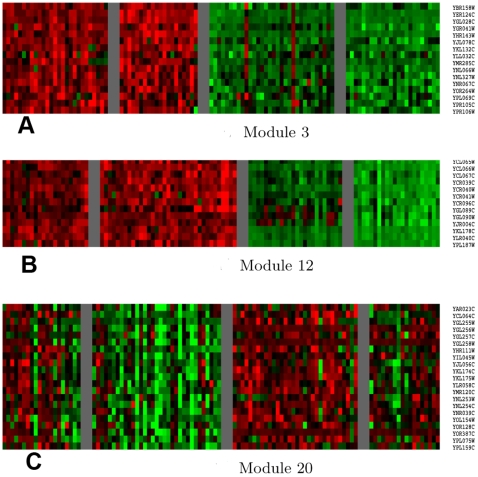
Heat map for expression of genes in modules. (A) for module 3; (B) for module 12; (C) for module 20. Each row represents a gene with the gene name listed on the right and each column represents an individual. Individuals are divided into four groups according to the genotypes of the two eQTLs. Over- and under-expression are indicated by red and green, respectively.

The interaction term for module 12 is statistically most significant (p-value = 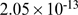). A previous study [16] experimentally validated that an interaction between *MAT* at the chromosome III locus and *GPA1* at the chromosome VIII locus affects a group of 19 genes. Among these 19 genes, one of them is not in our study set; two other genes were later experimentally verified to be “false positives” [16] and are correctly assigned to the null module in our analysis; and four other genes are negatively correlated with genes in this module and so are not placed in module 12. The remaining 12 genes are all recovered in this module. In addition, our method detects another gene, *HMLALPHA2*, which is also related to mating type. The heat map of the gene expression in module 12 is plotted in Figure 5B. This result demonstrates that our method not only is able to detect an experimentally validated interaction, but also has a higher specificity and sensitivity to detect the interaction than the regression based method.

Module 20 consists of 21 genes and is linked to two loci on chromosome XIII and X, respectively, but no epistatic interaction is detected between these loci. The heat map of the gene expression in this module is plotted in Figure 5C. Two transcription factor binding sites are enriched in the module, with the *ZAP1* binding site being the most significantly enriched (p-value = 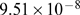). In fact, 14 of the 21 genes in module 20 are known or predicted to be *ZAP1* target genes [22] (p-value = 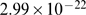). *ZAP1*, which is physically located at the chromosome X locus and has an eQTL at the chromosome XIII locus, is included in this module. A previously identified *ZAP1* module [17] overlaps significantly with module 20. Among the ten genes in the ZAP1 module, eight of them are also predicted in module 20. It was previously conjectured that a regulator at the chromosome XIII locus regulates *ZAP1* expression, and that as a result *ZAP1* expression and *ZAP1* genotype together affect *ZAP1* target genes [17]. Our model is consistent with this hypothesized mechanism and also identifies more *ZAP1* target genes in an objective way (i.e., regulators do not need to be pre-specified).

Module 3 is comprised of 16 genes and has the second most significant interaction term (p-value = 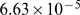). This module is linked to chromosomes II: 548401 and III: 177850. The heat map of the gene expression in this module is plotted in Figure 5A. Binding sites for *ACE2*, a transcription factor that activates expression of early G1-specific genes and that localizes to daughter cell nuclei after cytokinesis, are enriched in this module (p-value = 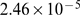). *AMN1*, a protein required for daughter cell separation and multiple mitotic checkpoints, is the only gene with a *cis*-eQTL in the module, and is predicted as at least one of the putative regulators for the eQTL hot spot at the chromosome II locus [10]–[11]. The *AMN1* allele swap signature [10] overlaps significantly with this module (p-value = 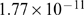). In addition, of the ten daughter-specific expression (DSE) genes identified in culture-averaged microarray experiments [23], nine are in our study set and seven of these are included in this module (p-value = 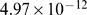). At the chromosome III locus is *BPH1*, a gene involved in cell wall organization. The RM version of *BPH1* has a deletion in the middle of the coding sequence compared to the BY sequence (Supplementary Figure S4), which results in an in-frame stop. Therefore, the RM version of *BPH1* may not be functional. When *BPH1* is knocked out, sporulation decreases [24]. However, we note that *BPH1* is in the null module, suggesting that the *BPH1* activity instead of its expression level may be linked to this locus.

**Figure 6:**
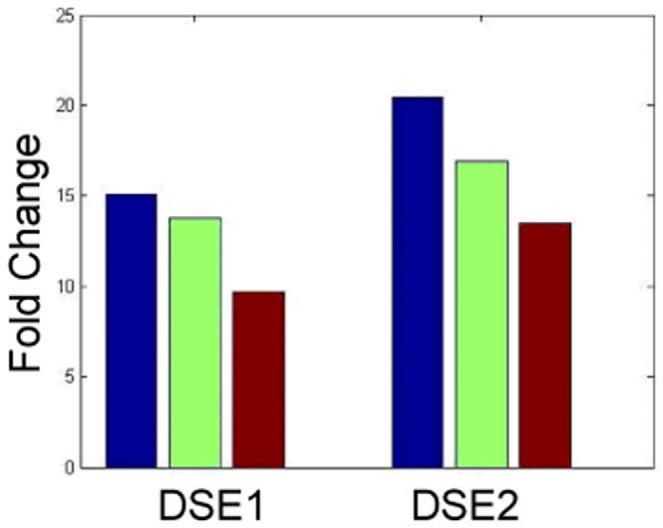
Comparison of the expression of *DSE1* and *DSE2* in different experiments. *DSE1* and *DSE2* are two daughter cell-specific genes in module 3. *DSE1* and *DSE2* are up-regulated 15.1- and 20.4-fold, respectively, in segregants bearing the BY allele at *AMN1* comparing to segregants bearing the RM allele at *AMN1* (blue bars). *DSE1* and *DSE2* are up-regulated 13.8- and 16.9-fold, respectively, in segregants bearing the BY allele at *AMN1* and the BY allele at *BPH1* comparing to segregants bearing the RM allele at *AMN1* and the BY allele at *BHP1* (green bars). *DSE1* and *DSE2* are up-regulated 9.7- and 15.3-fold, respectively, in the original BY strain relative to the engineered BY strain with RM allele at *AMN1* [25] (brown bars). It is clear that segregants categorized by both *AMN1* and *BPH1* alleles match the experimental result better.

To show that module 3 is under the regulation of two loci, we examined the expression of two genes in the module, *DSE1* and *DSE2*. *DSE1* and *DES2* are up-regulated 15.1- and 20.4-fold, respectively, in segregants carrying the BY allele at the *AMN1* locus relative to those carrying the RM allele. If we restrict attention to those segregants carrying the BY allele at the *BPH1* locus, *DES1* and *DES2* are up-regulated 13.8- and 16.9-fold, respectively, in segregants carrying the BY allele at the *AMN1* locus relative to those carrying the RM allele. When the RM version of *AMN1* was introduced onto the BY background, *DES1* and *DES2* were up-regulated only 9.7- and 13.5-fold in the BY wildtype compared to the BY engineered strain [25]. These results combined suggest that *AMN1* alone can not explain all of the variation in *DSE1* and *DSE2* expression, but the combination of the *AMN1* and *BPH1* alleles explains significantly more of the variation (shown in Figure 6).

## Discussion

We have developed a Bayesian partition model for simultaneously mapping multiple eQTLs for multiple sets of co-regulated genes. Whereas conventional linkage analysis has been widely and successfully applied to the study of one or a small number of traits at a time, our module-based method is suitable for analyzing thousands of phenotypes simultaneously. Both simulation studies and empirical data examples demonstrated that our method is effective for detecting marker interactions, even when no marginal effects could be detected. These improvements in power are a direct result of accounting for the correlation among gene expression traits and assessing the joint effect of multiple eQTLs, including interactions, on these correlated gene sets.

One of the main advances in our approach is the introduction of the “individual type” as a latent variable to describe associations between gene expression traits and markers. The individual type latent variable can be interpreted as a classification of individuals according to a combination of phenotypes and genotypes. The underlying mathematical model for this dependence structure is represented as a chain in which the joint distribution for some set of markers influences a set of expression traits via a latent “individual type” variable. After integrating out this latent variable, we observe a direct relationship between the marker and gene expression sets, similar to what would have been obtained from a the traditional regression model in the single-marker, single-gene case (Figures 2A and 2B). However, the advantage over the standard regression in introducing the latent individual type variable is its enabling us to model epistatic interactions and pleiotropy simultaneously.

Linkage disequilibrium (LD) among adjacent markers is an important feature of the genetic marker data. For individuals produced by the laboratory crosses (e.g., F1 and F2 designs), the marker dependency can be modeled satisfactorily by a Markov chain. The BP model can easily entertain this modification of the background marker distribution, but the computation time required to run this modified model dramatically increases since we need a forward-summation-backward-sampling algorithm to update the marker indicators (see *Supplemental Material* Text S1 for details). Another ad hoc strategy to account for the marker correlations without directly modeling them was to first scan all markers and to enumerate those marker pairs with correlations exceeding a given threshold. Then, in the MCMC algorithm, we imposed a mutually exclusive condition for such pairs so that highly correlated marker pairs would not appear simultaneously in any module.

We compared the Markov model approach with the ad hoc strategy on a small simulated data sets and a subset of the real data (data not shown). The *ad hoc* strategy always provided nearly identical results to that of the Markov model with only a fraction of the computation cost. Note that there are also markers that are highly correlated but are not physically linked [26]. In such cases the Markov model actually worked less satisfactorily than the ad hoc approach.

Our method shares some similarities to other methods in the literature, but also shows clear distinctions. For example, Lee *et al.* [17] proposed to simultaneously partition the gene expression and genotype markers. However, their method requires strong priors on the potential regulators, while our method does not. Kendzioski *et al.* [14] proposed a mixture of markers model to find the eQTLs for multiple gene expression. However, their method separates the gene clustering and eQTL mapping steps, where they first use k-means clustering to identify subsets of genes, and then apply eQTL mapping to the clusters of genes. In addition, their method does not address the epistatic effects. In contrast, gene expression partition and eQTL mapping are modeled jointly in our Bayesian method, and we are able to effectively detect epistasis by using a comprehensive statistical model on both the gene expression and the markers. Our analysis of the yeast data identified 20 modules linked to one eQTL and 9 modules linked to two eQTLs, among which three giving rise to strong epistatic interactions between markers. Some of the modules linked to two eQTLs are consistent with previously reported results [5],[17], and we were able to identify more true positive hits along with fewer false positives than previously reported.

It is of note that our approach can also be applied to mammalian data and to other quantitative traits data with discrete genetic and environmental covariates. In typical mouse studies, about 2000 SNPs are genotyped and 25,000 transcripts are measured, among which about 8000 are significantly differentially expressed [2]. The computation time will be at a similar order of the yeast data analysis. In typical human studies, 650,000 SNPs are genotyped and 40,000 transcripts are measured. The computation time will dramatically increase. We may, however, restrict our attention to hundreds of SNPs identified as possibly associated with gene expression traits in a human cohort, or/and to fewer expression traits identified as being relevant to diseases of interest [27]–[28]. In this type of scenarios, the input datasets would be roughly equivalent to the yeast data set described herein. Many other such applications can be imagined,

We are also improving parallelization implementation. Hopefully, we will be able to appropriately generalize and improve the Bayesian model as well as the MCMC algorithm so that our method can be applied to complete mammalian and other large data sets.

## Methods

### Bayesian partition model

A ***module*** is defined in the Results section as a set of gene expression traits (referred to as “genes” henceforth) and a set of genetic markers (e.g., SNPs) such that the mRNA expression variation of the genes is associated with the allelic variation of the markers. This association between multiple genes and markers is characterized by a latent indicator variable, individual type, conditional on which the trait and marker variables are independent of each other. The individual type latent variable can be viewed as representing a certain combination of markers that induces changes in expressions of a certain set of genes across different individual types.

To formally describe our model, consider a sample with *N* individuals. Each individual *i* is measured with *G* gene expression values denoted as 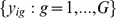 and *M* marker genotypes denoted as 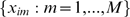. We assume that the observed data can be partitioned into *D* nontrivial modules plus a null component. The number of non-null modules, *D*, is pre-specified by the user and should reflect the user's prior belief in the higher level structure of the data. Every gene *g* or marker *m* belongs to one of the *D* nontrivial modules or the null module, determined by the gene indicator 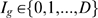 and the marker indicator 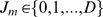. For each module 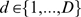, we further partition the *N* individuals into 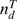 types denoted by the individual indicators 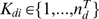 for 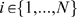. Each module may have a different number of individual types as well as different ways of partitioning the *N* individuals. For example, with a single biallelic marker (alleles ‘A’ and ‘a’) in the module, the module may have two individual types corresponding to genotypes aa vs. Aa or AA (dominant model), or 3 individual types corresponding to genotypes aa, Aa and AA (additive model). We seek module partitions in which expression patterns are similar for all genes, and gene expression variations across different individuals can be explained by the individual types. A cartoon illustration of the partition model is shown in Figure 1.

We model the gene expression traits in module *d* by an ANOVA model so that each trait value is the sum of the gene effect (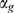), the eQTL effect for individual type *k* (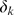), the individual effect (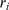), and an error term:
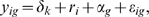
where gene *g* is in module *d*, *k* is the individual type of *i*, and *r_i_* and *α_g_* are *random effects*, following independent Gaussian distributions with mean zero.

To account for epistasis, we model the joint distribution of all the associated markers of module *d*, 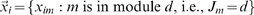, by a multinomial distribution, whose frequency vector is determined by the individual type *k*, i.e.,
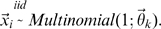
For example, if there are two markers 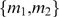 in the module and each has three genotypes, then there are nine combinations of the marker patterns. Thus 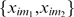 follows a 9-dimensional multinomial distribution.

For the null component, we assume that there is no association between the genes and the markers. The gene expression traits follow a normal distribution and the marker genotypes follow an independent multinomial distribution.

To avoid overfitting, we put an exponential prior on the indicator variables to penalize partitions with high complexity:
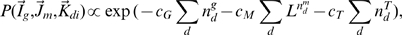
where 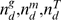 are the number of genes, markers and individual types in module *d*, and 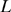 is the number of genotypes at each marker. We use conjugate priors on the continuous parameters, such as means and variances of the Gaussian distributions and frequency vectors of the multinomials, so that most of these parameters can be integrated out analytically to reduce the complexity of the posterior distribution.

The joint posterior distribution of all unknown variables is of the form:
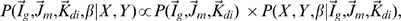
where *β* represents the set of left-over continuous parameters unable to be integrated out analytically. In order to make inference on the eQTL modules from this posterior distribution, we construct a Markov chain Monte Carlo method to traverse the joint space of all unknown parameters. Each Markov chain is randomly initialized, and uses the Gibbs sampler and the Metropolis-Hasting algorithm [18] to update the variables. We implement a split-merge algorithm, which is a special case of the reversible jump MCMC [29], to update the individual partitions globally. Parallel tempering [30] is used to help mixing the Markov chain. Further details of the modeling and sampling strategies can be found in the *Supplemental Material* Text S1.

Posterior probabilities are evaluated for each gene and candidate marker set to belong to each module based on the Monte Carlo samples. A threshold is then applied to the posterior probabilities to determine whether a particular gene and marker set should be included in a module.

### Application to the yeast data set

We assembled genotypic and expression data from 112 segregants obtained from a previously described yeast cross between the BY and RM strains of *S. cerevisiae* [15]. Of the 5,740 genes represented on the microarrays in this study, we selected 3,662 informative genes as input into the partition algorithm following the same criteria as previously described [10]. We then transformed the gene expression values by first performing quantile normalization [31] to make the distribution of the log-expression ratios for each individual to be the same, and then normalizing each gene so that the mean expression level for each gene was 0 and the standard deviation was 1.

Given that genes in the data set have been previously mapped to 13 distinct eQTL hot spots [11] and that there can be multiple causal factors for a single eQTL hot spot, we set the number of starting modules for our MCMC algorithm to 35∼45 (3×13 plus a null model) to account for these previously identified groups, and to also allow for the detection of new groups as well. For the parallel tempering implementation, we used 30 temperature ladders with almost equal spacing so that the average acceptance probability for exchanges between adjacent chains was roughly 0.15–0.3. We ran MCMC sampling for 1,000,000 iterations in each chain, which took one week of 30 CPUs (accounting for 30 parallel temperature ladders of the MCMC algorithm) on a Linux cluster with 2GHz CPUs. The log posterior probability and its auto-correlation curve depicted in Figures S5C and S5D highlight that the Markov chain became stationary after a burn-in period. See *Supplemental Material* Text S1 for more details.

Because markers in the yeast data set are very densely distributed, adjacent markers are almost always highly correlated. After MCMC sampling, markers adjacent to the “truly” linked marker often diluted the posterior probability for the true marker-module linkage. Since a proper Markov chain model for unlinked markers is computationally too expensive to implement (see *Supplemental Material* Text S1), we employed a heuristic method to counter this problem. We first specified a window centered at each marker so that markers inside the window are in high LD with the marker at the center. The posterior probabilities of all markers in the window were summed up and regarded as the modified posterior probability of the central marker. The markers with peak probabilities exceeding the given threshold were selected and all other markers in the corresponding windows were masked out.

Although we did not explicitly model pleiotropic effects for markers (i.e., single markers were not allowed to be associated with expression traits in multiple modules), we reported several modules mapped to the same markers in the yeast data set (See Table 3 and discussions in the *Supplemental Material* Text S1). The reason for this apparent contradiction is due to the aforementioned moving window approach and the dense distribution of the markers. In other words, if marker *m* is truly linked to two modules, in computation its adjacent markers can serve as its surrogates so that a subset of these markers are mapped to module 1, and the remainders mapped to module 2. Then the use of the moving window method can restore the total probability back to marker *m*.

To test the robustness of our result with respect to the initial parameters, we ran our program using three different numbers of modules, 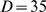, 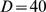 and 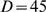, each having three independent runs. Samples from the run with the highest average posterior probability for each value of 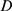 were used in the subsequent analyses. We chose 0.8 as the threshold for the posterior probabilities to determine the module membership for each gene and marker. We observed that more than 70% of the genes were consistently grouped together and mapped to the same markers (or null module) in all the runs with different *D* values. These genes and their associated markers formed the list of 29 modules.

## Supporting Information

Text S1 Supplementary methods and results
(0.49 MB PDF)

Figure S1 Module-by-module comparison of the Bayesian partition (BP) method and the step-wise regression (SR) method. (A) Number of the true positive gene-marker pairs detected in each module by the BP method (top) and the SR method (bottom). Nine different lines correspond to different posterior probability thresholds (for BP) or different FDR thresholds (for SR), both of which decrease from 0.9 to 0.1 linearly. There are 40 genes in each of the eight modules which are linked to two markers and thus the number of the true positive gene-marker pairs is 640. (B) Barplots of the number of true eQTLs detected in each module by the BP method (blue) and SR method (green). The shaded bar represents the number of genes detected as mapped to at least one of the true eQTLs while the solid bar represents the number of genes detected as mapped to both eQTLs. The thresholds are 0.5 for both posterior probability (BP) and FDR (SR). From Figure 1 we know that the total number of false positive gene-marker pairs is 11.41 and 38.04 for BP and SR respectively. When the thresholds are relaxed to 0.1, more eQTLs were detected in each category, as indicated by the vertical lines above the bars. However, the total number of the false positive gene-marker pairs is still lower using BP (178.37) compared to that using SR (267.07).
(0.36 MB TIF)

Figure S2 The distributions of LOD scores under the “single-gene-single-marker” model for genes in the 29 modules identified by the Bayesian method. (A) the LOD score distribution for genes in modules linked to a single eQTL. The LOD scores for 56.3% of transcripts were less than 4.35, the threshold corresponding to a genome-wide FDR of 0.01, and 11.5% of transcripts were less than 1.45, corresponding to a point-wise FDR of 0.01. (B) the LOD score distribution for genes in modules linked to two eQTLs. The LOD scores for 69% and 32.5% of transcripts were less than 4.35 and 1.45, corresponding to a genome-wide and a point-wise FDR of 0.01, respectively.
(0.11 MB TIF)

Figure S3 Plot of the causality test results for all pairs of genes between (A) module 4 and module 5 and (b) module 26 and 29. For a particular pair of genes (G1, G2) from module 4 and module 5, respectively, if the causality test claims that gene G1 is causal to gene G2 (corrected p-value<0.05), i.e. G1→G2, then a green dot is plotted at the corresponding position. Similarly, if the causality test results in G2→G1, then a red dot is plotted at the corresponding position. Genes in module 4 and module 5 are sorted for better visualization. Similar procedure applies to (B).
(0.34 MB TIF)

Figure S4 A local view of the coding sequence alignment of RM vs. BY for gene BPH1. The RM sequence has a deletion in the position labeled in red which results an in-frame stop.
(0.03 MB TIF)

Figure S5 Trace plots and autocorrelation plots of the log posterior probabilities for one of the simulated data set ((A) and (B)) and the yeast data set analysis ((C) and (D)). In (A), the trace plot was generated from two independent chains, each having 100,000 iterations, and the autocorrelation plot in (B) was obtained from the first chain at every 50 iterations. In (C), trace plot was generated from 1,000,000 Markov chain iterations, using D = 40 (D is the number of the modules). The last 700,000 iterations were used to generate the auto-correlation plot in (D).
(0.31 MB TIF)

Table S1 Design for the simulation II.
(0.20 MB PDF)

Table S2 True markers and inferred markers in each module.
(0.14 MB PDF)

Table S3 Enrichment of (A) gene knockout signatures and (B) TFBS for each module.
(0.15 MB PDF)
