# Supplementary material for: A Bayesian Partition Method for Detecting Pleiotropic and Epistatic eQTL Modules
Source: PLoS Comput Biol. 2010 Jan 15;6(1):e1000642. doi: 10.1371/journal.pcbi.1000642 (PMC2797600; doi:10.1371/journal.pcbi.1000642)
Supplement: Text S1 — Supplementary methods and results (0.49 MB PDF) [file pcbi.1000642.s001.pdf]

Supplemental Material for

A Bayesian Partition Method for Detecting Pleiotropic and Epistatic eQTL Modules

Wei Zhang[1], Jun Zhu[2,3], Eric E. Schadt[3,4] and Jun S. Liu[5§]

[1]UBS Equities, 677 Washington Blvd, Stamford, CT 06901
[2]Rosetta Inpharmatics, LLC, Merck & Co., Inc., 401 Terry Ave N, Seattle, WA 98109
[3]Sage Bionetworks, 1100 Fairview Ave N, Seattle, WA 98109
[4]Pacific Biosciences, 1505 Adams Drive, Menlo Park, CA 94025
[5]Department of Statistics, Harvard University, Cambridge, MA 02138

[§]Corresponding author. Email address: jliu@stat.harvard.edu

1. Bayesian partition model

Suppose we have a sample of N individuals. Each individual i is measured with G gene expression values denoted as $\{y_{ig} : g = 1,...,G\}$ and M marker genotypes denoted as $\{x_{im} : m = 1,...,M\}$. We partition the data into D modules $\{d : d = 1,...,D\}$ plus a null component $\{d : d = 0\}$ where the number of modules, D, is pre-specified. Each module d consists of n_d^G genes and n_d^M associated markers. Genes and markers that have no associations are partitioned into the null component. We further partition the N individuals into n_d^T types $\{t : t = 1,..., n_d^T\}$ with respect to each module d. Different modules may have a different number of individual types as well as different individual partitions. The overall partition of genes and markers into modules is determined by the gene indicators $\{I_g : g = 1,...N, I_g \in \{0,1,..,D\}\}$ and the marker indicators $\{J_m : m = 1,..., M, J_m \in \{0,1,....,D\}\}$, while the module-specific partition for individuals is determined by the individual indicators $\{K_{di} : d = 1,..., D, i = 1,...N, K_{di} \in \{1,..., n_d^T\}\}$.

In each module, the module genes and module markers are independently distributed conditional on the latent individual types. For genes in module d, we model their distributions as follows:

$$y_{ig} = \delta_k + r_i + \alpha_g + \varepsilon_{ig},$$

where gene *g* is in module *d* and *k* is the individual type of *i*; δ_k is the eQTL effect determined by the individual type $k = K_{di}$; r_i is the effect of other regulators, such as transcription factors; α_g explains the gene effect; and ε_{ig} is the random measurement error. All genes in the same module share the same eQTL effect and individual effect, the combination of which, denoted as $\beta_{di} = \delta_k + r_i$, can be viewed as the module center. We put a normal-inverse-chi-square distribution on $\{\delta_k, r_i, \alpha_g, \varepsilon_{ig}\}$:

$$r_i \overset{iid}{\sim} N(0, \sigma_d^2),$$

$$\delta_k \mid \sigma_d^2 \overset{iid}{\sim} N(0, \sigma_d^2 / k_\sigma), \;\; \sigma_d^2 \sim Inv-\chi^2(v_\sigma, s_\sigma^2),$$

$$\varepsilon_{ig} \overset{iid}{\sim} N(0, \tau_d^2),$$

$$\alpha_g \mid \tau_d^2 \overset{iid}{\sim} N(0, \tau_d^2 / k_\tau), \;\; \tau_d^2 \sim Inv-\chi^2(v_\tau, s_\tau^2),$$

where $(k_\sigma, v_\sigma, s_\sigma^2; k_\tau, v_\tau, s_\tau^2)$ are the hyper parameters for the conjugate normal priors.

To account for epistasis, we model the joint distribution of all the associated markers in a module, denoted as $\vec{x}_i = \{x_{im} : m \text{ is in module } d, \text{ i.e. } J_m = d\}$, by a multinomial distribution whose frequency parameters are determined by the individual type $k = K_{di}$. We also put a conjugate prior distribution on these parameters:

$$\vec{x}_i \overset{iid}{\sim} Multinomial(1; \vec{\theta}_k), \;\; \vec{\theta}_k = \{\theta_k^1, ..., \theta_k^{L^{n_d^M}}\},$$

$$\vec{\theta}_k \sim Diri(\vec{\alpha}_k), \;\; \alpha_k^1 = \alpha_k^2 = ... = \alpha_k^{L^{n_d^M}} = \frac{\lambda}{L^{n_d^M}},$$

where $\vec{\theta}_k$ is the frequency vector of the multinomial distribution for the individual type *k* in module *d*; $\vec{\alpha}_k$ is the hyper parameters for $\vec{\theta}_k$; L is the number of possible genotypes at each marker; $n_d^M = \sum_{m:J_m=d}$ is the total number of linked markers in module *d*; and λ is the pseudo-count for the Dirichlet prior. For example, if there are two markers in the module and the genotype at each marker can take any of the three possible values, then there are potentially $L^{n_d^M} = 3^2 = 9$ combinations of marker genotypes. In this case,

$\vec{\theta}_k = \{\theta_k^1, ..., \theta_k^9\}$ are the frequencies for each of the 9 configurations and

$\vec{\alpha}_k = \{\alpha_k^1, ..., \alpha_k^9\} = \{\frac{\lambda}{9}, ..., \frac{\lambda}{9}\}$, representing that these 9 configurations are equally likely in the prior.

For the null component, we assume that there is no association between the genes and the markers. Each gene expression trait follows a normal distribution and each marker follows an independent multinomial distribution. The models for the expression and the markers are as follows.

$$y_{ig} = \alpha_g + \varepsilon_{ig}, g \in \{g' : I_{g'} = 0\},$$

$$\varepsilon_{ig} \overset{iid}{\sim} N(0, \tau_0^2),$$

$$\alpha_g \mid \tau_0^2 \overset{iid}{\sim} N(0, \tau_0^2 / k_0), \quad \tau_0^2 \sim Inv-\chi^2(v_0, s_0^2),$$

$$x_{im} \overset{iid}{\sim} Multinomial(1; \vec{\theta}_m),\ m \in \{m' : J_{m'} = 0\},$$

$$\vec{\theta}_m \sim Diri(\vec{\alpha}_m), \quad \alpha_m^1 = \alpha_m^2 = ... = \alpha_m^L = \frac{\lambda_0}{L}.$$

We then have the following explicit expression:

$$\begin{aligned}
P(X, Y, \vec{\beta}_{di} \mid \vec{I}_g, \vec{J}_m, \vec{K}_{di}, \Theta) = & \underbrace{\prod_{g:I_g=0} (2\pi\tau_0^2)^{-N/2} \exp\{-\frac{1}{2\tau_0^2}\sum_{i=1}^{N}(y_{ig} - \alpha_g)^2\}}_{\text{genes in the NULL component}} \\
& \times \underbrace{\prod_{m:J_m=0} \vec{\theta}_m^{\vec{\#}_m}}_{\text{markers in the NULL component}} \\
& \times \prod_{d=1}^{D} \underbrace{\prod_{g:I_g=d} (2\pi\tau_d^2)^{-N/2} \exp\{-\frac{1}{2\tau_d^2}\sum_{i=1}^{N}(y_{ig} - \alpha_g - \beta_{di})^2\}}_{\text{genes in the module } d} \\
& \times \prod_{d=1}^{D}\prod_{k=1}^{n_d^T} \underbrace{\vec{\theta}_k^{\vec{\#}_k}}_{\text{markers in the individual type } k \text{ of module } d} \\
& \times \prod_{d=1}^{D}\prod_{k=1}^{n_d^T} \underbrace{(2\pi\sigma_d^2)^{-n_{dk}^I/2} \exp\{-\frac{1}{2\sigma_d^2}\sum_{i:K_{di}=k}(\beta_{di} - \delta_k)^2\}}_{\text{module center in the individual type } t \text{ of module } d},
\end{aligned}$$

where $\vec{\#}_m$ denotes the observed genotype count vector for marker *m* and $\vec{\#}_k$ denotes the observed genotype count vector for markers in module *d* among individuals with type *k*.

For example, if we observe 20 individuals having genotype “aa”, 40 individuals having genotype “aA” and 25 individuals having genotype “AA” for marker *m*, then $\vec{\#}_m = \{20, 40, 25\}$. n_d^T is the number of individual types in module *d*; and n_{dk}^I is the number of individuals with type *k* in module *d*.

The prior distributions for the indicators are assumed to be as the following:

$$P(\vec{I}_g, \vec{J}_m, \vec{K}_{di}) \propto \exp\{-c_G \sum_{d=1}^{D} n_d^G - c_M \sum_{d=1}^{D} L^{n_d^M} - c_T \sum_{d=1}^{D} n_d^T\}$$

where n_d^G, n_d^M, n_d^T are the number of genes, markers and individual types in module *d*. c_G, c_M and c_T are pre-fixed parameters to penalize partitions with high complexity. The full posterior distribution is

$$P(\vec{I}_g, \vec{J}_m, \vec{K}_{di}, \vec{\beta}_{di}, \Theta \mid X, Y) \propto P(\vec{I}_g, \vec{J}_m, \vec{K}_{di}) P(\Theta \mid \vec{I}_g, \vec{J}_m, \vec{K}_{di}) P(X, Y, \vec{\beta}_{di} \mid \vec{I}_g, \vec{J}_m, \vec{K}_{di}, \Theta)$$

Updating the parameters with a large dimension is nontrivial. In addition, we do not fix the number of individual types, so that when a new individual type (*k*) is generated, we need to generate the corresponding parameters for the eQTL effect (δ_k) and genotype frequency vector ($\vec{\theta}_k$). Although it is possible to propose new parameters using the reversible jumping rule, the acceptance rate is usually very low. Here we adopt a more effective alternative using the idea of predictive updating [1]. With this updating scheme we integrate out all the parameters from their prior distributions and get the marginal posterior distribution for the indicators, which is the product of the following:

$$P(\vec{I}_g,\vec{J}_m,\vec{K}_{di},\vec{\beta}_{di}\mid X,Y)\propto P(\vec{I}_g,\vec{J}_m,\vec{K}_{di})\int P(\Theta\mid \vec{I}_g,\vec{J}_m,\vec{K}_{di})P(X,Y,\vec{\beta}_{di}\mid \vec{I}_g,\vec{J}_m,\vec{K}_{di},\Theta)d\Theta$$

Prior $$\propto \exp\{-c_G\sum_{d=1}^{D} n_d^G - c_M\sum_{d=1}^{D} L^{n_d^M} - c_T\sum_{d=1}^{D} n_d^T\}\times$$

Markers in the NULL module $$\prod_{m:J_m=0}\frac{\Gamma(\lambda_0)}{\Gamma(\lambda_0+N)}\prod_{l=1}^{L}\frac{\Gamma(\lambda_0/L+\#_m^l)}{\Gamma(\lambda_0/L)}\times$$

Genes in the NULL module $$(\frac{\kappa_0}{\kappa_0+N})^{n_0^G/2}\frac{\Gamma(\frac{\nu_0+Nn_0^G}{2})(\nu_0 s_0^2)^{\nu_0/2}}{\Gamma(\frac{\nu_0}{2})(\nu_0 s_0^2+SS_0)^{(\nu_0+Nn_0^G)/2}}\times$$

Markers in the module $$\prod_{d=1}^{D}\prod_{k=1}^{n_d^T}\frac{\Gamma(\lambda)}{\Gamma(\lambda+n_{dk}^I)}\prod\frac{\Gamma(\lambda/L^{n_d^M}+\vec{\#}_k)}{\Gamma(\lambda/L^{n_d^M})}\times$$

Genes in the module $$\prod_{d=1}^{D}(\frac{\kappa_\tau}{\kappa_\tau+N})^{n_d^G/2}\frac{\Gamma(\frac{\nu_\tau+Nn_d^G}{2})(\nu_\tau s_\tau^2)^{\nu_\tau/2}}{\Gamma(\frac{\nu_\tau}{2})(\nu_\tau s_\tau^2+SS_d)^{(\nu_\tau+Nn_d^G)/2}}\times$$

.

Module center $$\prod_{d=1}^{D}\frac{\Gamma(\frac{\nu_\sigma+N}{2})(\nu_\sigma s_\sigma^2)^{\nu_\sigma/2}}{\Gamma(\frac{\nu_\sigma}{2})(\nu_\sigma s_\sigma^2+SS_{d\beta})^{(\nu_\sigma+N)/2}}\prod_{k=1}^{n_d^T}\sqrt{\frac{\kappa_\sigma}{\kappa_\sigma+n_{dk}^I}}$$

Here,

$$SS_0=\sum_{g:I_g=0}\left\{\sum_{i=1}^{N}y_{ig}^2-\frac{1}{k_0+N}(\sum_{i=1}^{N}y_{ig})^2\right\},$$

$$SS_d=\sum_{g:I_g=d}\left\{\sum_{i=1}^{N}(y_{ig}-\beta_{di})^2-\frac{1}{k_\tau+N}(\sum_{i=1}^{N}(y_{ig}-\beta_{di}))^2\right\},$$

$$SS_{d\beta}=\sum_{i=1}^{N}\beta_{di}^2-\sum_{k=1}^{n_d^T}\frac{1}{k_\sigma+n_{dk}^I}(\sum_{i:K_{di}=k}\beta_{di})^2 .$$

2. Modeling considerations

Several parameters need to be specified in our model, including the number of modules D, the penalty parameters (c_G, c_M, c_T), the hyper parameters for the modules $(k_\tau,\nu_\tau,s_\tau^2;k_\sigma,\nu_\sigma,s_\sigma^2;\lambda)$ and the hyper parameters for the null component $(k_0,\nu_0,s_0^2,\lambda_0)$. The module size D is determined based on the prior information about the data set. In simulations, we found that as long as D is as large as or larger than the true number of

modules in the data set, the algorithm can always detect module genes and their linked markers. What happens when D is larger than the true number of modules in the data is that some modules appear to be empty (either with no genes or with no markers) because the posterior probability does not exceed the threshold. Thus in practice we need to choose the module size D large enough based on the prior information in order to recover all the modules in the data. Through simulation studies, we found that the result is not sensitive to the penalty parameters (c_G, c_M, c_T) over a certain range. While the posterior probability changes a lot if we change these parameters, the final configuration remains approximately the same. More specifically, the rankings of the genes and markers in each module based on the posterior distribution are quite stable over a large range of the penalty parameters. The choices of $k_0, v_0, \lambda_0, k_\tau, v_\tau, \lambda$ also do not significantly affect the final configuration, mostly because the information coming from the data outweighs the information from the prior. Given this stability, for simplicity we use parameter values $k_0 = v_0 = \lambda_0 = 1, k_\tau = v_\tau = \lambda = 1$. We choose s_0^2 as the empirical estimate of the variance of gene expression and set $s_\tau^2 = s_0^2 / 2$. The magnitude of k_σ controls the prior information for the eQTL effect δ_k. A smaller value corresponds to a wider spread and thus allows for more heterogeneity in the components and tends to favor configurations with a smaller number of individual types. We use $k_\sigma = 10^{-6}$ to maintain the model simplicity. The magnitude of v_σ controls the strength of the prior information for the variance of δ_k. A larger value of v_σ together with a smaller value of s_σ^2 tends to favor tighter clusters of individual types. Due to the small sample size in our study (~100 individuals), we use $v_\sigma = N/3$ and $s_\sigma^2 \approx s_0^2/100$.

3. Computation strategy

Basic MCMC moves

An exhaustive evaluation of all possible partition configurations is infeasible due to its ultra-high cardinality. Therefore, we construct a Markov chain to traverse the joint space of all possible partitions and module centers whose unique stationary distribution is

the measure of interest. Starting the Markov chain from a randomly selected point, we iterate sampling with the following seven types of moves:

1. Sweep of the gene indicators. For each gene *g* in turn, we sample I_g from its conditional posterior distribution $P(I_g \mid \vec{I}_{-g}, \vec{J}_m, \vec{K}_{di}, X, Y, \vec{\beta}_{di})$. In other words, we calculate $p_d = P(I_g = d \mid \vec{I}_{-g}, \vec{J}_m, \vec{K}_{di}, X, Y, \vec{\beta}_{di})$ for $d = 0,1,...,D$ and assign the gene *g* into module d with probability p_d.
2. Sweep of the marker indicators. For each marker *m* in turn, we sample J_m from its conditional posterior distribution $P(J_m \mid \vec{I}_g, \vec{J}_{-m}, \vec{K}_{di}, X, Y, \vec{\beta}_{di})$. In other words, we calculate $p_d = P(J_m = d \mid \vec{I}_g, \vec{J}_{-m}, \vec{K}_{di}, X, Y, \vec{\beta}_{di})$ for $d = 0,1,...,D$ and assign the marker *m* into module d with probability p_d.
3. Sweep of the individual indicators. For each module *d* and each individual *i*, we sample K_{di} from its conditional posterior distribution $P(K_{di} \mid \vec{I}_g, \vec{J}_m, \vec{K}_{-di}, X, Y, \vec{\beta}_{di})$. In other words, we calculate $p_k = P(K_{di} = k \mid \vec{I}_g, \vec{J}_m, \vec{K}_{-di}, X, Y, \vec{\beta}_{di})$ for $k = 1,...,n_d^T$. If possible, we also calculate the probability that individual *i* belongs to a new individual type $k = n_d^T + 1$. Then we assign the individual *i* into type *k* with probability p_k. Special attention is paid when we generate a singleton type or when we remove a singleton type.
4. Metropolis move of the module center. For each module *d* and each individual *i*, we propose a change from β_{di} to $\beta_{di} + \Delta_{di}$ conditional on all the other parameters. The proposal is accepted according to the metropolis ratio.
5. Exchange of individual indicators. For each module *d*, we randomly select two individuals in different individual types and propose to exchange their indicators. The metropolis ratio is calculated to determine whether we accept the exchange or not.
6. Exchange a module marker with its adjacent marker. For each module in turn, we randomly select one of the module markers and propose to exchange its indicator with its adjacent marker indicator. This metropolis step accounts for linkage

disequilibrium between adjacent markers.

7. Exchange a module marker with a marker in the null component. For each module in turn, we randomly select one of the module markers. We then randomly select one of the markers in the null component and propose to switch their indicators.

Parallel tempering

It has been observed that the MCMC samplers can be very sticky, especially when dealing with a large number of discrete variables. To help mixing, we implement parallel tempering [2]. Suppose the target distribution is $\pi(x)$. We construct a temperature ladder $1 = t_1 < t_2 < ... < t_R$ and define $\pi_r(x) \propto \pi(x)^{1/t_r}$. The sampler space of the parallel tempering is the product space of π_r. The new target distribution is

$$\pi(x_1, x_2, ..., x_R) \propto \pi_1(x_1)\pi_2(x_2)..\pi_R(x_R)$$

The parallel tempering process consists of the following steps:

1. Iterative sampling at each level $r = 1,..,R$.
2. For every N_0 (say 100) cycles of updating, propose a cycle of swaps starting from the highest temperature level. For $r = R-1, ..., 1$, calculate the metropolis ratio

$$\alpha = \min\left\{1, \frac{\pi_r(x_{r+1})\pi_{r+1}(x_r)}{\pi_r(x_r)\pi_{r+1}(x_{r+1})}\right\} = \min\left\{1, \left(\frac{\pi(x_{r+1})}{\pi(x_r)}\right)^{1/t_r - 1/t_{r+1}}\right\}.$$

Then we swap x_r and x_{r+1} with probability α.

Global move of the individual indicators – splitting and merging

Although we allow the individual types to vary across different modules, the chain of the individual indicators is still very sticky given that we update one individual indicator conditional on all the others. To address this problem we add two types of global moves to facilitate transversal of the MCMC chain: splitting and merging, which are special cases of reversible jump schemes [3]. In splitting, an individual type *k* with more than two individuals in a given module *d* is selected at random. A proposal of splitting it into two types is considered and then the Metropolis ratio is calculated to determine whether to accept the change or not. Because there are many ways to split an

individual type into two, many of the splits will be too random and so have no chance of getting accepted. Therefore, as a first step we run a two-means clustering algorithm on the module center $\{\beta_{di} : K_{di} = k\}$ and extract the means (μ_1, μ_2) and the pooled variance of the two clusters. Each individual *i* is then assigned to either cluster according to a normal distribution on β_{di}, whose mean and variance are estimated as above. To guarantee reversibility, we check two types of consistency for a valid proposal. First, if the two estimated means are $\mu_1 \le \mu_2$, then we ensure that the two sample means of the module center after the proposed splitting satisfy $\bar{\beta}_{k1} \le \bar{\beta}_{k2}$. Second, we sort the sample means of the module center before splitting as $\bar{\beta}_1 \le ... \le \bar{\beta}_k \le ... \bar{\beta}_{n_d^T}$. After splitting, we require that the new sample means satisfy $\bar{\beta}_1 \le ... \le \bar{\beta}_{k1} \le \bar{\beta}_{k2} \le ... \bar{\beta}_{n_d^T}$. For the merging move, we first sort the sample means of the module center as $\bar{\beta}_1 \le ... \le \bar{\beta}_{n_d^T}$. Then we randomly select two adjacent groups and propose to merge them into one. These two types of MCMC moves greatly improve the mixing of the MCMC chains.

Dimension change when updating the marker indicators

To improve the sampling efficiency, we set a lower bound on the number of module markers during the burn-in period, and we gradually reduce the bound to one before the burn-in period ends. Because the number of multi-marker combinations increases exponentially as more markers are introduced, it is difficult to sample large number of interactions when starting with a small set of markers, unless the marker set in the current iteration contains partial interactions that have detectable effects. Therefore, it is advantageous to include a large number of markers at the beginning of the process as this will increase our chances of sampling true interacting markers. In simulation studies, we found that this approach effectively detected epistasis within a limited number of sampling steps.

Forward summation for dependent markers

Suppose there are *L* markers on chromosome 1, each having two genotype values 0/1. Previously we modeled that the markers in the "null module" are mutually independent. To accommodate linkage disequilibrium among closely spaced markers, we

assume here that the genotypes of these L markers follow a first order inhomogeneous Markov chain with the transition function $f_t(i,j) \equiv P(M_{t+1} = j \mid M_t = i)$. When no marker is linked to any module, it is easy to write down the joint probability of the L markers on the chromosome as

$$P(M_1 = m_1, M_2 = m_2, \dots, M_L = m_L) = p_1(m_1) \prod_{t=1}^{L-1} f_t(m_t, m_{t+1}),$$

where $p_1(m)$ is the allele frequency of the first marker.

However, if one or more markers are linked to some non-null modules, we have to model the linked markers using our module model previously described, and the joint probability of those unlinked markers needs to be conditioned on the values of the linked markers. More previously, suppose marker M_k is the only marker on this chromosome that is currently linked to a module, i.e. $J_k = 1$. Let $M_{[-k]}$ denote all markers but k. Then,

$$P\left(M_{[-k]} \middle| M_k = l\right) = \frac{p_1(m_1) \prod_{t=1}^{L-1} f_t(M_t, M_{t+1})}{P(M_k = l)}.$$

The denominator is

$$P(M_k = l) = \sum_{m_{k-1}=0}^{1} \cdots \sum_{m_1=0}^{1} \{p_1(m_1) \times f_1(m_1, m_2) \times f_2(m_2, m_3) \times \cdots \times f_{k-1}(m_{k-1}, l)\},$$

This sum can be computed recursively:

$$g_{t+1}(m) = \sum_{l=0}^{1} g_t(l) f_t\,(l, m), \qquad t = 1, \dots, k,$$

with $g_1(m) = p_1(m)$.

Now, we assume that two markers M_k and M_j (with $k < j$) are linked with some module(s), with marker values $M_k = l_1$ and $M_j = l_2$, and the remaining markers are unlinked. Then, we need to compute $P\left(M_k = l_1, M_j = l_2\right)$ recursively by summing up the first k-1 marker variables, and then marker variables from $k+1$ to $j-1$. Again, starting with $g_1(m) = p_1(m)$, we compute

$$g_{t+1}(m) = \sum_{l=0}^{1} g_t(l) f_t(l, m),\ t = 1, \dots, k-1.$$

Then, we define $g_{k+1}(l_1, m) = g_k(l_1) f_k(l_1, m)$, and start another recursion:

$$g_{s+1}(l_1, m) = \sum_{l=0}^{1} g_s(l_1, l) f_s(l, m),$$

for $s = k+1, \ldots, j-1$. Finally, $g_j(l_1, l_2)$ is our desired $P(M_k = l_1, M_j = l_2)$.

With the above calculations, we can update each marker in turn to see if it should be linked to a certain module. For example, suppose at the current iteration marker M_k is already linked to a certain module. Then, the odds for another marker M_j to be linked to the same module versus not linked to any module would be

$$\frac{P(M_{[-k,-j]}|M_k, M_j)P_{module}(M_k, M_j, \text{others})}{P(M_{[-k]}|M_k)P_{module}(M_k, \text{others})} = \frac{P(M_k)P_{module}(M_k, M_j, \text{others})}{P(M_k, M_j)P_{module}(M_k, \text{others})},$$

where P_{module} represents the probability model for the markers when they are linked to certain modules, and $P(\ )$ are the probability distributions under the null Markov chain model.

Missing data

The problem of missing data is easily handled in the Bayesian framework by considering a joint distribution on an expanded parameter space that includes both the indicator variables and missing data. MCMC samplers then iterates between sampling the indicator variables conditional on the imputed missing data, as described above in the absence of missing data, and sampling the values for the missing data conditional on the indicator variables. This latter step draws the missing data from its predicted distribution. For example, if the value of a gene expression of an individual is missing, its predicted distribution given the indicator variables is a t-distribution. If the value of a marker genotype in an individual is missing, its predicted distribution given the indicator variables is a multinomial distribution whose frequency parameters are proportional to the sums of the pseudo counts in the prior and observed counts in the remaining data. In practice, we impute the missing data using some imputation techniques, such as *k*-nearest neighbor imputation, before applying our module algorithm to reduce the computation load.

4. Simulation design

We simulated 120 individuals with 500 binary markers equally spaced on 20 chromosomes, each of length 100 cM, using the "*qtl*" package in R. This type of simulated population is similar in structure to previously described segregating yeast

populations [4]. We constructed eight eQTL modules, each consisting of 40 gene traits and two associated eQTLs positioned at marker loci. An additional 680 gene expression were randomly generated from a standard Gaussian distribution independently, giving rise to a total of 1000 gene expression traits.

For each of the eight modules, we first simulated a "core gene" according to the corresponding regression model and the proportion of phenotypic variance attributable to the eQTLs [5], as depicted in Table 1(a). The heritability is defined as the fraction of variance in segregant phenotypes attributable to genetic factors. In each model, $e \sim N(0, \sigma_e^2)$ represents the environmental noise. The regression coefficient β in each model is chosen so that the heritability, $h^2 = (\sigma_s^2 - \sigma_p^2)/\sigma_s^2$, is 0.6, where σ_s^2 and σ_p^2 are the variances among phenotype values in the segregants and the pooled variance among parental measurements, respectively. Because the variance in the parental measurements reflects only measurement error, in the simulation we set $\sigma_p^2 = \sigma_e^2 = 1$. For example, in module B, where $R = \beta I_{x1=x2} + e$, it is easy to see that $\sigma_s^2 = \beta^2/4 + 1$, thus we are able to solve for the value of $\beta = \pm\sqrt{6}$. Given the "core gene", 40 gene expression traits were then generated independently from a Gaussian model so that the average correlation of these genes with the "core gene" was 0.5. The procedure was repeated independently 100 times.

After simulation, we calculated the percentage of variation explained by the true model in a module. For example, for each gene in module B we calculated the sum of squares of the gene expression for all 120 samples (SS_{total}) and the residual sum of squares within the two sample groups: those with $x_1 = x_2$ and those with $x_1 \neq x_2$ (SS_{res}). The percentage of variation explained by the model for this gene is $1 - SS_{res}/SS_{total}$. This value was calculated for all 40 genes in module B, and the average is listed in the third column of Table 1A.

5. Results of the simulation study and graphical display

We analyzed the simulated data sets using two methods: (1) our Bayesian partition method using parallel tempering [6] with 15 temperature ladders and 100,000 MCMC

iterations each, referred to as BP; (2) the two-stage regression method proposed by Storey *et al* [7], referred to as SR. The trace and auto-correlation plots for one simulated data set, shown in Figures S5A and S5B, demonstrate that the Markov chain used in our method attained a stationary distribution after the burn-in period. SR is a special application of the step-wise regression. In the first stage, SR identifies the most significant marker for each gene expression trait based on the one-gene-one-marker regression model. It then proceeds to find the next most significant marker conditional on the previous detected marker for each gene. Permutation tests over all genes are carried out in each stage to control the overall FDR.

To get a better understanding of the signal strength in each module, we divided the total genetic variance for a two-locus model into three components: the genetic variance at locus 1, the genetic variance at locus 2, and the epistatic (or interaction) variance using the classical analysis of variance [8-10]. In Table 1B, each row displays the genotype at the first locus, and each column indexes the genotype at the second locus. Each cell contains a genotypic mean and its respective frequency in parentheses. Given the genotypic means and frequencies at both loci, one can calculate the mean (μ) and the total genetic variance (σ^2):

$$\mu = p_{AB}\mu_{AB} + p_{Ab}\mu_{Ab} + p_{aB}\mu_{aB} + p_{ab}\mu_{ab}$$

$$\sigma^2 = p_{AB}(\mu_{AB} - \mu)^2 + p_{Ab}(\mu_{Ab} - \mu)^2 + p_{aB}(\mu_{aB} - \mu)^2 + p_{ab}(\mu_{ab} - \mu)^2$$

The amounts of genetic variance at locus 1 and locus 2 are:

$$\sigma_1^2 = p_A(\mu_A - \mu)^2 + p_a(\mu_a - \mu)^2$$

$$\sigma_2^2 = p_B(\mu_B - \mu)^2 + p_b(\mu_b - \mu)^2,$$

where

$$\mu_A = (p_{AB}\mu_{AB} + p_{Ab}\mu_{Ab})/(p_{AB} + p_{Ab}), \mu_a = (p_{aB}\mu_{aB} + p_{ab}\mu_{ab})/(p_{aB} + p_{aB})$$

$$\mu_B = (p_{AB}\mu_{AB} + p_{aB}\mu_{aB})/(p_{AB} + p_{aB}), \mu_b = (p_{Ab}\mu_{Ab} + p_{ab}\mu_{ab})/(p_{Ab} + p_{ab})$$

The epistatic variance is defined as the amount of genetic variance not accounted for by the single-locus components. We calculated the average percentage of the single-locus variances and the epistatic variance for each module. These values are listed in the last three columns of Table 1A of the main text.

We compared the total number of the true gene-marker pairs detected in each module at various thresholds (Figure S1B). As expected, the SR method had a high failure rate when the marginal effects of both markers are weak, even at a very generous threshold. This can be seen in modules B, D, and H where no or very weak marginal effect is present and genetic variations are mainly explained by the epistasis. In modules E, F, and G where the major marker explains more than 70% of the genetic variation, the SR method detected the major marker in nearly 50% of the simulations at the 0.5 threshold, but not the minor marker (Figure S1B). Thus, the total numbers of the true gene-marker pairs detected by SR in these modules were only about 20 out of 80. In modules A and C where the marginal effects of the two marker are almost the same, the SR method detected one of the markers for some genes, but the detection rates were lower than those in modules E, F and G (Figure S1B) because neither marker has a very strong marginal effect. In contrast, the BP method performed superiorly in all eight modules.

For a graphical illustration of the BP analysis, we simulated another dataset consisting of 120 individuals measured with 1000 genes and 500 markers. Given the haploid nature of the segregants, 500 binary markers are equally spaced on 20 chromosomes, each of length 100cM, using the "*qtl*" package in R. We simulated four modules, A, B, C, D, each containing 60, 60, 40, and 40 genes, which are associated with 3, 2, 1 and 2 markers, respectively. The associated markers are randomly selected and do not overlap. To mimic the inter-correlation of the genes in real gene expression data, we first generated a core gene *R* in each module according to the corresponding models depicted in Table S1. In each model, $e \sim N(0, \sigma_e^2)$ represents the environmental noise. The regression coefficient β in each model is determined by the corresponding heritability, which is defined as $h^2 = (\sigma_s^2 - \sigma_p^2)/\sigma_s^2$, where σ_s^2 and σ_p^2 are the variances of the phenotype values in the segregants and the pooled parental measurements, respectively. Because the variance in the parental measurements reflect only measurement error, we set $\sigma_p^2 = \sigma_e^2 = 1$. For example, in module B, $\sigma_s^2 = \beta^2/4 + 1$, $h^2 = 0.7$, we thus have $\beta = \pm\sqrt{28/3}$. After generating the core gene, we simulated the gene expression traits in each module from a Gaussian model where the average correlation to the core gene is set as in Table S1 and genes in the same module are

independent conditional on the core gene. Finally, as in the previous simulation example, we calculated the percentage of variation explained by the true model averaged over all genes in a module, and listed in the last column of Table S1.

As shown in Figure 4 of the main text, all of the genes in the null component were correctly classified. Most genes in the other four modules were also correctly classified. To find the linked markers in each module, we not only counted the marginal number of appearances for each marker in each module but also the number of joint appearances in order to account for the joint effect. The truly linked markers and the posterior inference are summarized in Table S2. We see that the truly linked markers were correctly identified for modules A, B and D. In module B, our method picked the true marker pair (490, 149) and marker pair (491, 149) with about 0.5 probabilities each. This is due to strong linkage between makers 490 and 491. In module C, our method correctly picked the true linked marker, 292, but then also picked up some false positive markers, albeit with a small probability. In all cases, our method correctly identified the truly associated markers with high posterior probabilities.

6. Inferring causal relationships between expression traits

For two expression traits T_1 and T_2 linked to the same locus L in the yeast cross, there are four possible basic relationships as previously described[11].

- Causal model from T_1 to T_2. DNA variations at locus L lead to changes in trait T_1 and that in turn lead to changes in trait T_2 ($L \rightarrow T_1 \rightarrow T_2$);
- Causal model from T_2 to T_1. DNA variations at locus L lead to changes in trait T_2 and that in turn lead to changes in trait T_1 ($L \rightarrow T_2 \rightarrow T_1$);
- Conditional independent model. DNA variations at locus L independently lead to changes in traits T_1 and T_2 ($T_1 \leftarrow L \rightarrow T_2$);
- The complete model ($T_1 \leftarrow L \rightarrow T_2$).

Given two modules associated with a common locus, the causality test is applied to all possible gene pairs between the two modules. The p-value for declaring a causal relationship is $0.05/(0.5 * n_{d1} * n_{d2})$, where n_{d1} and n_{d2} are sizes of the two modules.

8. eQTL modules in yeast

We applied our Bayesian method to the yeast data and obtained a list of 29 modules. Among these 29 modules, 20 are linked to a single eQTL while the remaining nine are linked to two eQTLs. Three of the nine linking to two eQTLs give rise to significant epistatic interactions between the two loci. For modules linked to a single eQTL, the distribution of LOD scores for single transcripts at the associated markers is shown in the Supplementary Figure S2A. We note that the LOD scores for 56.3% of transcripts were smaller than 4.35, the threshold corresponding to a genome-wide FDR of 0.01; the LOD scores for 11.5% of transcripts were smaller than 1.45, corresponding to a point-wise FDR of 0.01. These results highlight that greater than 50% of the marker-transcript associations in the single marker modules cannot be identified by simple pairwise analysis. Even though some marker-transcript associations are weak, most transcripts in each module are significantly correlated and share coherent GO biological processes as we show below. Because the Bayesian partition method leverages the covariance information in a module, it has qa greater power to detect true associations. In addition, the Bayesian partition method can group transcripts linked to the same marker into multiple modules that may represent different causal factors (as was highlighted for modules 1, 2 and 4) or causal/reactive relationships between modules (as was highlighted for modules 26 and 28).

For double-marker modules, the distribution of LOD scores for transcripts associated with the markers corresponding to the modules is shown in the Supplementary Figure S2B. The LOD scores for 69% and 32.5% of the transcripts at the associated markers were smaller than 4.35 and 1.45, respectively, thresholds corresponding to a genome-wide FDR of 0.01 and point-wise FDR of 0.01, respectively. Given that there are more transcript-marker associations (69%) for double-markers modules that are smaller than the 4.35 LOD score threshold, compared to 56.3% for single-marker modules, these results likely reflect the generally weaker transcript-marker associations at the second markers. Only 10% (15 out of 151) of the transcripts in the double-marker module were linked to both markers with LOD scores above 4.35. These results suggest

that most of the transcript-marker associations in the double-marker modules cannot be found by focusing only on the strong associations.

We tested the enrichment of these modules using GO terms, Rosetta knockout compendium [12] and transcription factor binding sites [13]. The results are listed in Table S1.

Module 1 consists of 38 genes which are mapped to chromosome II: 548401, No GO term, knockout signature, or TF binding site is enriched in the module. It does not overlap with any of the 13 eQTL hot spots.

Module 2 consists of 33 genes which are mapped to chromosome II: 548401. There is neither GO term nor TF binding site enriched in the module. SHE4 knockout signature is enriched in the module (corrected p-value= 0.022). It overlaps with eQTL hot spot 2 (corrected p-value= 7.47×10^{-11}).

Module 3 consists of 16 genes that are mapped to chromosome II: 548401 and chromosome III: 177850. The GOCC cell wall is enriched in the module (corrected p-value= 1.5×10^{-4}). Six gene knockout signatures and three TF binding sites are enriched in the module, listed in Table S1. Binding sites for ACE2, a transcription factor that activates expression of early G1-specific genes, localizes to daughter cell nuclei after cytokinesis, are enriched in the module (corrected p-value= 2.46×10^{-5}). It overlaps with eQTL hot spots 2 and 3 (corrected p-value= 1.24×10^{-10} and 0.0035). The genetic structure based on a regression model of the first principal component of this module is

$$\mathrm{Y=-3.5016+5.5841*M1-0.6669\times M2+1.9564\times M1:M2},$$

where $\mathrm{M1}$ and $\mathrm{M2}$ are genotypes at the linked markers 1 and 2, respectively. The p-values for the first marker, the second marker and their interactions are 2×10^{-16}, 0.0672 and 6.63×10^{-5}, respectively, suggesting strong interaction between the two loci. Two candidate regulators AMN1 and BPH1 are located at these two loci. Details are described in the main text.

Module 4 consists of 137 genes that are linked to chromosome II: 548401. This module is enriched for genes with GOCC nucleolus (corrected p-value= 7.84×10^{-73}). Nine gene knockout signatures are enriched in the module, including RPL27A, RPL8A, BUD21 and BUD22. No TF binding site is enriched in the module. Using the *de novo*

motif finding algorithm AlignACE [14], we found that this module is highly enriched with the motif elements PAC (GCGATGAGATG, MAP score=389.081) and RRPE (TGAAAAATTT, MAP score=308.639), which have been previously conjectured to be interacting in the regulation of the rRNA transcription genes [15-16].

Module 5 consists of 75 genes that are linked to chromosome II: 548401. There is neither GO term nor gene knockout signature enriched in the module. MBP1's binding sites are enriched in the module (corrected p-value= 0.0096). AlignACE was also applied to this module. The PAC (MAP score=34.573) and RRPE (MAP score=33.917) motifs are much less enriched in this module compared to module 4.

Module 6 contains 38 genes that are mapped to chromosome II: 602012. GOBF protein disulfide isomerase activity is enriched in this module (corrected p-value = 5.26×10^{-4}). Two gene knockout signatures and one TF binding site are enriched in the module.

Module 7 consists of 83 genes that are mapped to chromosome III: 79091 and chromosome XV: 170945. GOBP 'de novo' IMP biosynthesis is enriched in the model (corrected p-value= 3.17×10^{-4}). There are 17 gene knockout signatures and two TF binding sites enriched in the module including GCN4 knockout signature and GCN4 binding site. This module overlaps with eQTL hot spot 4 on chromosome III, hot spots 10 and 12 on chromosome XV. The genetic structure based on a regression model of the first principal component of this module is

$$Y=-0.1969 - 4.5798\times M1+5.0811\times M2-0.7319\times M1{:}M2 .$$

The p-values for the first marker, the second marker and their interaction are 0.000487, 9.70×10^{-5} and 0.70, respectively. The model suggests that the two eQTLs have an additive effect on this module but no interaction effect. ILV6 is the only gene with cis-eQTL at chromosome III locus in this module. There is no gene with cis-eQTL on chromosome XV in this module.

Module 8 consists of 69 genes that are linked to chromosome III: 79091. GOBP histidine biosynthesis is enriched in the module (corrected p-value= 0.01). There are 53 gene knockout signatures and two TF binding sites enriched in the module.

Module 9 consists of 61 genes that are linked to chromosome III: 79091. There is neither GO term nor TF binding site enriched in the module. Seven gene knockout signatures are enriched in the module.

Module 10 consists of 18 genes which are linked to chromosome III: 81832. GOBP branched chain family amino acid biosynthesis is enriched in the module (corrected p-value=1.76×10^{-3}). 18 gene knockout signatures are enriched in the module. LEU3 binding site is enriched in the module (corrected p-value=1.07×10^{-9}).

Module 11 consists of 52 genes which are linked to chromosome III: 81832 and chromosome VIII: 84437. GOCC nuclear chromosome is enriched in this module (corrected p-value=4.41×10^{-9}). Three gene knockout signatures and two TF binding sites are enriched in the module. The genetic structure based on a regression model of the first principal component of this module is

$$\text{Y=0.7310+3.3015}\times\text{M1-4.4392}\times\text{M2+1.3355}\times\text{M1:M2}.$$

The p-values for the first marker, the second marker, and their interaction are 0.00481, 1.74×10^{-5}, and 0.37, respectively. The interaction between the two loci is not significant.

Module 12 consists of 13 genes that are linked to chromosome III: 201166 and chromosome VIII: 111679. The GOBP regulation of transcription from RNA polymerase II promoter is enriched in the module (corrected p-value=0.0094). One gene knockout signature is enriched in the module. There is no TF binding site enriched in the module. The genetic structure based on a regression model of the first principal component of this module is

$$\text{Y=-2.5886+4.3786}\times\text{M1-0.7028}\times\text{M2+3.08}\times\text{M1:M2}.$$

The p-values for the first marker, the second markers and their interaction are 2×10^{-16}, 0.00791 and 2.05×10^{-13}, respectively. The interaction between the two loci is significant. Previous study [17] has experimentally validated that the interaction between MAT on chromosome III locus and GPA1 on chromosome VIII locus affects this group of genes.

Module 13 consists of 9 genes that are linked to chromosome III: 201166. There is no GO term enriched in this module. Ten gene knockout signatures three TF binding sites are enriched in the module. This module is negatively correlated with the module 12 ($r=-0.77$).

Module 14 consists of 13 genes that are linked to chromosome V: 116530. GOBP 'de novo' pyrimidine base biosynthetic process is enriched in the module (corrected p-value$=3.55\times10^{-4}$). Four gene knockout signatures are enriched in the module. No TF binding site is enriched.

Module 15 consists of 44 genes that are linked to chromosome VIII: 111690. GOCC mating projection tip is enriched in the module (corrected p-value$=7.44\times10^{-8}$). Twenty gene knockout signatures and three TF binding sites are enriched in the module, including knockout signatures and TF binding sites for both DIG1 and STE12.

Module 16 consists of 10 genes that are mapped to c chromosome X: 22315 and chromosome VI: 28041. GOBP aldehyde metabolism is enriched in the module (corrected p-value$=1.16\times10^{-8}$). Neither gene knockout signature nor TF binding site is enriched in the module. The genetic structure based on a regression model of the first principal component of this module is

$$\text{Y=-0.7776-4.1667}\times\text{M1+1.5344}\times\text{M2+1.7623}\times\text{M1:M2}.$$

The p-values for the first marker, the second marker and their interaction are 2×10^{-16}, 7.04×10^{-5} and 0.00097, respectively. The interaction between the two loci is significant. AAD10, a putative aryl-alcohol dehydrogenase, is a cis-gene linked to the chromosome X locus. Six of the 10 genes in the module share similar sequences of AAD6 and AAD16, which are physically located at the chromosome VI locus. The result suggests that the two loci and their interaction are due to cross-hybridization.

Module 17 consists of 11 genes that are linked to chromosome XII: 659357 and chromosome XIII: 430164. There is neither GO term nor TF binding site enriched in the module. Twelve gene knockout signatures are enriched in the module. The genetic structure based on a regression model of the first principal component of this module is

$$\text{Y=-0.1592+2.8327}\times\text{M1-2.1231}\times\text{M2-0.1164}\times\text{M1:M2}.$$

The p-values for the first marker, the second marker and their interaction are 5.2×10^{-5}, 8.49×10^{-4} and 0.90, respectively. Ten of 11 genes share similar sequences as PAU genes. It is likely that this module is due to cross hybridization.

Module 18 consists of 45 genes that are linked to chromosome XII: 662627 and chromosome III: 79091. GOBP ergosterol biosynthesis is enriched in the module

(corrected p-value$=1.9\times10^{-28}$). Six gene knockout signatures are enriched in the module. HAP1 binding site is enriched in the module (corrected p-value$=1.1\times10^{-23}$). The genetic structure based on a regression model of the first principal component of this module is

$$Y=2.4752-7.72042\times M1+3.2330\times M2-2.1490\times M1:M2.$$

The p-values for the first marker, the second marker, and their interaction are 2×10^{-16}, 1.93×10^{-7}, and 0.0148, respectively. The regression model indicates that the major part of the genetic variation comes from the marginal effects of the two eQTLs. This module significantly overlaps with the eQTL hot spot 8 where HAP1 was predicted as the causal regulator [18]. However, HAP1 itself is in the noise module due to its negative correlation with the module genes (average correlation is -0.48). Even though HAP1 is not in this module, the module is not only enriched for genes with HAP1 binding site, but also is enriched for the HAP1 knockout signature (p-value$=1.19\times10^{-31}$). Thus, HAP1 is the causal regulator at the first locus. It is worth to note that the second eQTL on chromosome III is also a major hot spot for amino acid metabolic process. Although the effect of the second eQTL is strong (p-value$=1.93\times10^{-7}$) in the above model, the marginal effect is weak (p-value$=0.00316$) without conditional on the first eQTL, as

$$Y=-1.2605+2.8235\times M2.$$

Module 19 consists of 34 genes that are linked to chromosome XII: 1056097 and chromosome IV: 1525327. GOBP telomerase-independent telomere maintenance is enriched in the model (corrected p-value$=4.2\times10^{-10}$). Eleven gene knockout signatures are enriched in the module. No TF binding site is enriched. The genetic structure based on a regression model of the first principal component of this module is

$$Y=-4.0217+5.2371\times M1+3.5512\times M2-1.6520\times M1:M2.$$

The p-values for the first marker, the second marker and their interaction are 2.98×10^{-9}, 8.4×10^{-6} and 0.134. The regression model indicates that the genetic variation is explained mainly by the marginal effects of the two eQTLs. YRF1-4, YRF1-5, and YLR462W are physically located at the first locus; YRF1-1 and YER189W are at the second locus. Twenty-five genes in the module share high sequence similarity to YRF1-1 and YRF1-4. The rest nine genes are at telomeres and also share sequence similarities among themselves (YPR203W, YFL065C and YLR462 are similar; YEL075C,

YER189W, YFL064C and YPR202W are similar). YRF1 genes are found in telomeric Y' elements and encode a DNA helicase. YRF1 genes are induced in survivors defective for telomerase [19]. Main perturbation may be telomere length variation at chromosomes IV and XII between the two strains. Then, cross-hybridizations give rise to the complex genetics of the module.

Module 20 consists of 21 genes that are linked to chromosome XIII: 49903 and chromosome X: 327852. No GO term is enriched in the module. Four gene knockout signatures are enriched in the module. Binding sites for two TFs BAS1 and ZAP1 are enriched (corrected p-value= 0.046 and 9.51×10^{-8}, respectively). The genetic structure based on a regression model of the first principal component of this module is

$$\text{Y=-0.5528+3.4727}\times\text{M1-1.9648}\times\text{M2-1.1304}\times\text{M1:M2}.$$

The p-values for the first marker, the second marker and their interaction are 1.14×10^{-5}, 0.011 and 0.27, respectively. ZAP1 binding site is enriched in the module. Previous research [20] has identified a similar module and hypothesized that a regulator at chromosome XIII locus regulates the expression of ZAP1, and then ZAP1 expression and ZAP1 genotype together affect ZAP1 target genes.

Module 21 consists of 81 genes that are linked to 4XIV: 449639. The GOCC endoplasmic reticulum is enriched in the module (corrected p-value= 9.68×10^{-7}). Two gene knockout signatures are enriched in the module. No TF binding site is enriched in the module.

Module 22 consists of 52 genes that are linked to chromosome XIV: 486861. The GOBF structural constituent of ribosome is enriched in the module (corrected p-value = 1.17×10^{-32}). Two gene knockout signatures are enriched in the module. No TF binding site is enriched in the module.

Module 23 consists of 68 genes that are mapped to chromosome XIV: 486861. The GOCC Arp2/3 protein complex is enriched in the module (corrected p-value = 8.10×10^{-4}). Neither gene knockout signature nor TF binding site is enriched in the module.

Module 24 consists of 39 genes that are linked to chromosome XIV: 449639. The GOBP nuclear pore organization and biogenesis is enriched in the module (corrected p-

value= 0.007). Neither gene knockout signature nor TF binding site is enriched in the module.

Module 25 consists of 77 genes that are linked to chromosome XIV: 486861. The GOCC mitochondrial inner membrane is enriched in the module (corrected p-value = 5.12×10^{-5}). No knockout signature is enriched in the module.

Module 26 consists of 83 genes that are linked to chromosome XV: 170945. The GOBP response to stress is enriched in the module (corrected p-value= 1.99×10^{-7}). Thirty-three gene knockout signatures are enriched in the module. MSN4 binding site is enriched in the module (corrected p-value= 3.83×10^{-4}).

Module 27 consists of 45 genes that are linked to chromosome XV: 170945. No GO term, knockout signature or TF binding site is enriched in the module.

Module 28 consists of 74 genes that are linked to chromosome XV: 170945. The GOBF fructose transporter activity is enriched in the module (corrected p-value= 0.0092). Four knockout signatures are enriched in the module. No TF binding site is enriched in the module.

Module 29 consists of 42 genes that are linked to chromosome XV: 563943. The GOCC respiratory chain complex III is enriched in the module (corrected p-value = 7.9×10^{-12}). Ten gene knockout signatures are enriched in the module. Binding sites for five TFs (HAP1-5) are enriched in the module.

8. Multiple modules linked to complex eQTL hot spots in yeast

Although we did not explicitly model pleiotropic effects for markers (i.e., single markers were not allowed to be associated with expression traits in multiple modules), we are able to link multiple modules to the same markers as described in the Methods section. Several modules are linked to loci that correspond to previously identified eQTL hot spots [18]. These modules are either causally/reactively related with respect to the linked locus, or they are linked to the same locus due to the short physical distance between the target markers. For example, modules 4 and 5 are tightly correlated. Genes in module 5 are less correlated with each other (average correlation=0.404) than genes in module 4 (average correlation = 0.716), and their LOD scores with respect to the chromosome II locus are on average smaller than the LOD scores for genes in module 4. To further

explore the relationship between module 4 and module 5, we carried out a causality test [11] to all gene pairs between these two modules. As shown in Figure S3 (a), among the 137 genes in module 4, 117 are "causal" (corrected p-value<0.05) to one or more genes in module 5, while only four out of the 75 genes in module 5 are causal to one or more genes in module 4. This suggests that module 4 may reflect the primary effect and module 5 a secondary response.

Modules 7 to 11 are linked to the same locus on chromosome III, and modules 7-10 significantly overlap with the eQTL hot spot 4 (Table 2). It was previously demonstrated that LEU2 and ILV6 are causal regulators for this hot spot [21]. It is of particular note that LEU2 has been placed in the null module because it is negatively correlated with the genes in modules 7-10. In fact, the average of the correlation coefficients for LEU2 expression and genes in modules 7-11 are -0.264, -0.361, -0.271, -0.644, and 0.219, respectively. On the other hand, ILV6 was placed in module 7. The LEU2 knockout signature overlaps significantly with modules 8 (p-value= 2.19×10^{-14}) and 10 (p-value= 1.36×10^{-8}) but not modules 7, 9 and 11, while the ILV6 knockout signature overlaps significantly with modules 7-10 (p-value= 8.78×10^{-12}, 3.61×10^{-13}, 2.65×10^{-6}, and 3.57×10^{-9}, respectively). These results suggest that there are multiple causal regulators for this eQTL hot spot and that our method is sensitive enough to dissect the difference.

Modules 26-28 are linked to a locus on chromosome XV that is coincident with eQTL hot spot 12, with all modules significantly overlapping with genes linked to this locus (p-value = 1.08×10^{-10}, 3.11×10^{-11}, and 9.01×10^{-11}, respectively). The average intra-module correlation for module 26 (0.731) is higher than that for modules 27 (0.409) and 28 (0.459). *PHM7* was previously identified and validated as a causal regulator for this hot spot [21]. The *PHM7* knockout signature significantly overlaps with modules 26 and 28 (p-value= 8.93×10^{-5} and 0.0016, respectively). When compared to a previously constructed yeast knockout compendium [12], module 26 overlaps with 33 knockout signatures, while module 28 overlaps with only four of the knockout signatures (three of the four also overlap with module 26). Application of a causality procedure [11] revealed that 52 genes (out of 83) in module 26 were supported as causal for at least one gene in module 28, while only six genes (out of 74) in module 28 were supported as causal for at least one gene in module 26 (Figure S3 (b)). These results indicate that genes in module

26 serve as the primary response to the causal perturbation of *PHM7* and genes in module 28 serve as the secondary response. Other causal regulators for module 27 that are independent of *PHM7* may exist.

References:

1. Liu JS (1994) The collapsed gibbs sampler with application to a gene regulation problem. J Am Statistit Ass 89: 958-966.
2. Geyer CJ (1991) Markov chain Monte Carlo maximum likelihood. Computing Science and Statistics. Fairfax: Interface Foundations. pp. 156-163.
3. Green PJ (1995) Reversible jump Markov chain Monte Carlo computation and Bayesian model determination. Biometrika 82: 711-732.
4. Brem RB, Yvert G, Clinton R, Kruglyak L (2002) Genetic dissection of transcriptional regulation in budding yeast. Science 296: 752-755.
5. Lander ES, Botstein D (1989) Mapping mendelian factors underlying quantitative traits using RFLP linkage maps. Genetics 121: 185-199.
6. Liu JS (2001) Monte Carlo strategies in scientific computing. New York: Springer.
7. Storey JD, Akey JM, Kruglyak L (2005) Multiple locus linkage analysis of genomewide expression in yeast. PLoS Biol 3: e267.
8. Cockerham CC (1954) An extension of the concept of partitioning hereditary variance for analysis of covariances among relatives when epistasis is present. Genetics 39: 859-882.
9. Fisher RA (1918) The correlation between relatives on the supposition of Mendelian inheritance. Trans R Soc Edinb 52: 399-433.
10. Tiwari HK, Elston RC (1997) Deriving components of genetic variance for multilocus models. Genet Epidemiol 14: 1131-1136.
11. Schadt EE, Lamb J, Yang X, Zhu J, Edwards S, et al. (2005) An integrative genomics approach to infer causal associations between gene expression and disease. Nat Genet 37: 710-717.
12. Hughes TR, Marton MJ, Jones AR, Roberts CJ, Stoughton R, et al. (2000) Functional discovery via a compendium of expression profiles. Cell 102: 109-126.
13. MacIsaac KD, Wang T, Gordon DB, Gifford DK, Stormo GD, et al. (2006) An improved map of conserved regulatory sites for Saccharomyces cerevisiae. BMC Bioinformatics 7: 113.
14. Hughes JD, Estep PW, Tavazoie S, Church GM (2000) Computational identification of cis-regulatory elements associated with groups of functionally related genes in Saccharomyces cerevisiae. J Mol Biol 296: 1205-1214.
15. Tanay A, Regev A, Shamir R (2005) Conservation and evolvability in regulatory networks: the evolution of ribosomal regulation in yeast. Proc Natl Acad Sci U S A 102: 7203-7208.
16. Beer MA, Tavazoie S (2004) Predicting gene expression from sequence. Cell 117: 185-198.
17. Brem RB, Storey JD, Whittle J, Kruglyak L (2005) Genetic interactions between polymorphisms that affect gene expression in yeast. Nature 436: 701-703.

18. Yvert G, Brem RB, Whittle J, Akey JM, Foss E, et al. (2003) Trans-acting regulatory variation in Saccharomyces cerevisiae and the role of transcription factors. Nat Genet 35: 57-64.
19. Yamada M, Hayatsu N, Matsuura A, Ishikawa F (1998) Y'-Help1, a DNA helicase encoded by the yeast subtelomeric Y' element, is induced in survivors defective for telomerase. J Biol Chem 273: 33360-33366.
20. Lee SI, Pe'er D, Dudley AM, Church GM, Koller D (2006) Identifying regulatory mechanisms using individual variation reveals key role for chromatin modification. Proc Natl Acad Sci U S A 103: 14062-14067.
21. Zhu J, Zhang B, Smith EN, Drees B, Brem RB, et al. (2008) Integrating large-scale functional genomic data to dissect the complexity of yeast regulatory networks. Nat Genet 40: 854-861.
