# Supplementary figures and images for: A Bayesian Partition Method for Detecting Pleiotropic and Epistatic eQTL Modules
Source: PLoS Comput Biol. 2010 Jan 15;6(1):e1000642. doi: 10.1371/journal.pcbi.1000642 (PMC2797600; doi:10.1371/journal.pcbi.1000642)

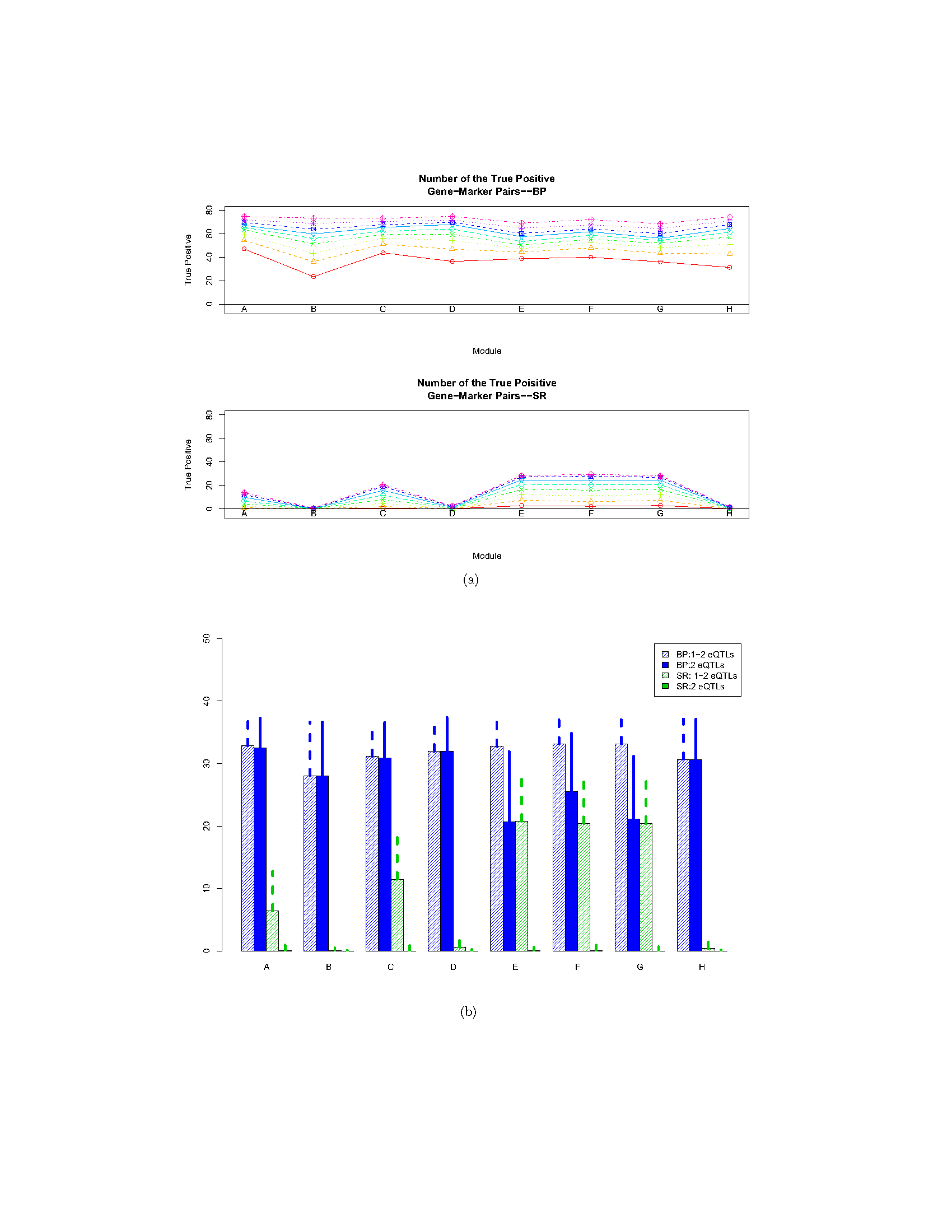

Supplement: Figure S1 — Module-by-module comparison of the Bayesian partition (BP) method and the step-wise regression (SR) method. (A) Number of the true positive gene-marker pairs detected in each module by the BP method (top) and the SR method (bottom). Nine different lines correspond to different posterior probability thresholds (for BP) or different FDR thresholds (for SR), both of which decrease from 0.9 to 0.1 linearly. There are 40 genes in each of the eight modules which are linked to two markers and thus the number of the true positive gene-marker pairs is 640. (B) Barplots of the number of true eQTLs detected in each module by the BP method (blue) and SR method (green). The shaded bar represents the number of genes detected as mapped to at least one of the true eQTLs while the solid bar represents the number of genes detected as mapped to both eQTLs. The thresholds are 0.5 for both posterior probability (BP) and FDR (SR). From Figure 1 we know that the total number of false positive gene-marker pairs is 11.41 and 38.04 for BP and SR respectively. When the thresholds are relaxed to 0.1, more eQTLs were detected in each category, as indicated by the vertical lines above the bars. However, the total number of the false positive gene-marker pairs is still lower using BP (178.37) compared to that using SR (267.07). (0.36 MB TIF) [file pcbi.1000642.s002.tif]

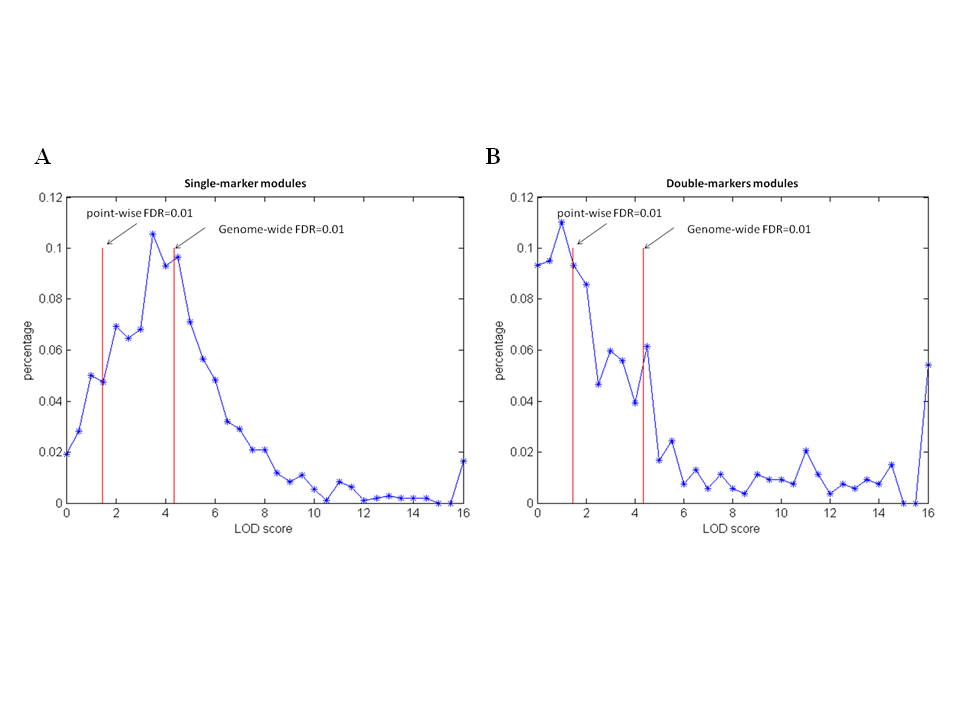

Supplement: Figure S2 — The distributions of LOD scores under the “single-gene-single-marker” model for genes in the 29 modules identified by the Bayesian method. (A) the LOD score distribution for genes in modules linked to a single eQTL. The LOD scores for 56.3% of transcripts were less than 4.35, the threshold corresponding to a genome-wide FDR of 0.01, and 11.5% of transcripts were less than 1.45, corresponding to a point-wise FDR of 0.01. (B) the LOD score distribution for genes in modules linked to two eQTLs. The LOD scores for 69% and 32.5% of transcripts were less than 4.35 and 1.45, corresponding to a genome-wide and a point-wise FDR of 0.01, respectively. (0.11 MB TIF) [file pcbi.1000642.s003.tif]

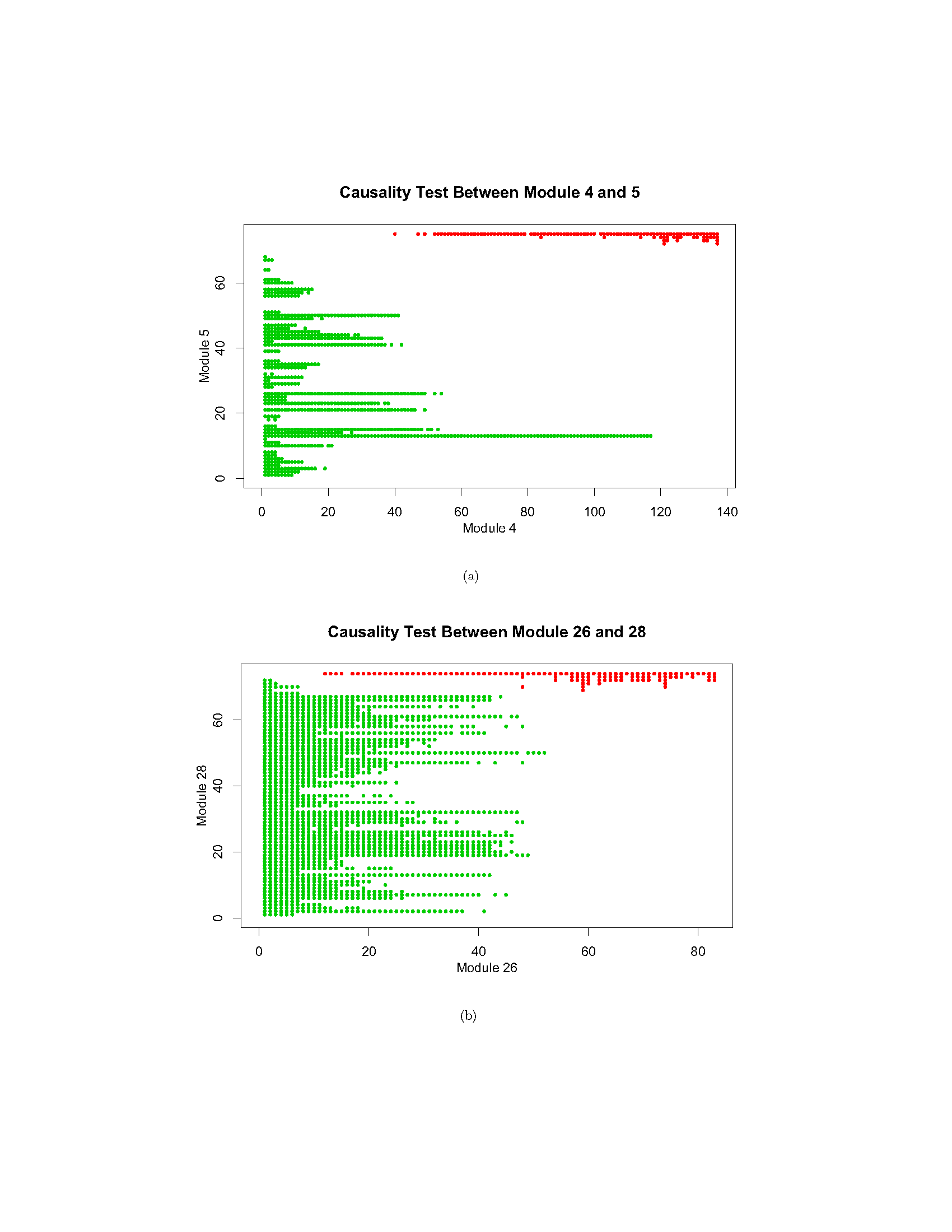

Supplement: Figure S3 — Plot of the causality test results for all pairs of genes between (A) module 4 and module 5 and (b) module 26 and 29. For a particular pair of genes (G1, G2) from module 4 and module 5, respectively, if the causality test claims that gene G1 is causal to gene G2 (corrected p-value<0.05), i.e. G1→G2, then a green dot is plotted at the corresponding position. Similarly, if the causality test results in G2→G1, then a red dot is plotted at the corresponding position. Genes in module 4 and module 5 are sorted for better visualization. Similar procedure applies to (B). (0.34 MB TIF) [file pcbi.1000642.s004.tif]

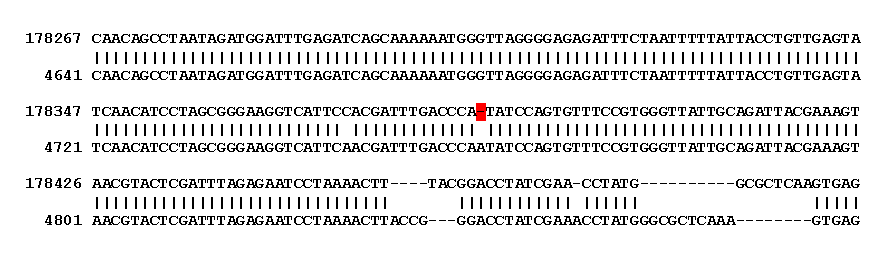

Supplement: Figure S4 — A local view of the coding sequence alignment of RM vs. BY for gene BPH1. The RM sequence has a deletion in the position labeled in red which results an in-frame stop. (0.03 MB TIF) [file pcbi.1000642.s005.tif]

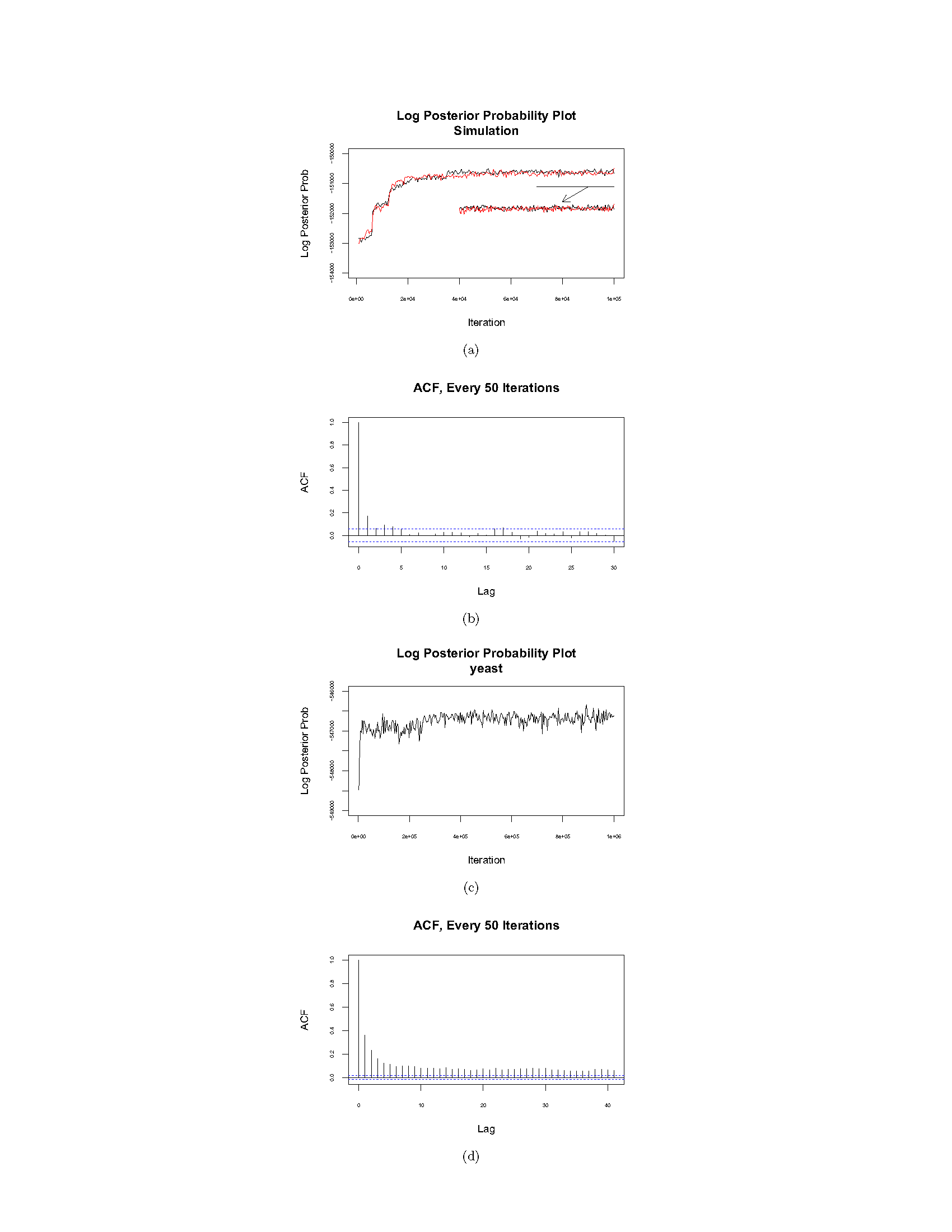

Supplement: Figure S5 — Trace plots and autocorrelation plots of the log posterior probabilities for one of the simulated data set ((A) and (B)) and the yeast data set analysis ((C) and (D)). In (A), the trace plot was generated from two independent chains, each having 100,000 iterations, and the autocorrelation plot in (B) was obtained from the first chain at every 50 iterations. In (C), trace plot was generated from 1,000,000 Markov chain iterations, using D = 40 (D is the number of the modules). The last 700,000 iterations were used to generate the auto-correlation plot in (D). (0.31 MB TIF) [file pcbi.1000642.s006.tif]
