# Supplementary material for: A Bayesian Partition Method for Detecting Pleiotropic and Epistatic eQTL Modules
Source: PLoS Comput Biol. 2010 Jan 15;6(1):e1000642. doi: 10.1371/journal.pcbi.1000642 (PMC2797600; doi:10.1371/journal.pcbi.1000642)
Supplement: Table S1 — Design for the simulation II. (0.20 MB PDF) [file pcbi.1000642.s007.pdf]

Table S1: Design for the simulation II. [a]: Regression models used to simulate the core genes. We denote x_i^d as the ith marker in module *d*. [b]: Number of genes in the module. [c]: Heritability of the core gene. [d]: Average correlation of the genes in the module with the core gene. [e]: Average percentage of variations for genes in the module explained by the true model.

Module	Model[a]	#Genes[b]	Heritability[c]	Cor.[d]	% of Var.[e]
A	$R=\beta I_{x_1^A=x_2^A}+\beta I_{x_3^A=1}+e$	60	0.85	0.5	23.66
B	$R=\beta I_{x_1^B=x_2^B}+e$	60	0.7	0.5	18.8
C	$R=\beta I_{x_1^C=1}+e$	40	0.65	0.5	15.6
D	$R=\beta I_{x_1^D=1}+\beta I_{x_2^D=1}+e$	40	0.7	0.5	18.6
