# Supplementary material for: A Bayesian Partition Method for Detecting Pleiotropic and Epistatic eQTL Modules
Source: PLoS Comput Biol. 2010 Jan 15;6(1):e1000642. doi: 10.1371/journal.pcbi.1000642 (PMC2797600; doi:10.1371/journal.pcbi.1000642)
Supplement: Table S2 — True markers and inferred markers in each module. (0.14 MB PDF) [file pcbi.1000642.s008.pdf]

Table S2: True markers and inferred markers in each module. Posterior probabilities are calculated based on the joint appearance of the corresponding marker(s) from the last 25,000 iterations.

Module	True Markers	Posterior Inference	
		Markers	Posterior Prob.
A	(270, 100, 172)	(270, 100, 172)	0.988
B	(490, 149)	(490, 149)	0.503
		(491, 149)	0.490
C	292	292	0.751
		(292, 61)	0.142
		(292, 62)	0.103
D	(443, 191)	(443, 191)	0.813
		191	0.109
