# Supplementary material for: A Bayesian Partition Method for Detecting Pleiotropic and Epistatic eQTL Modules
Source: PLoS Comput Biol. 2010 Jan 15;6(1):e1000642. doi: 10.1371/journal.pcbi.1000642 (PMC2797600; doi:10.1371/journal.pcbi.1000642)
Supplement: Table S3 — Enrichment of (A) gene knockout signatures and (B) TFBS for each module. (0.15 MB PDF) [file pcbi.1000642.s009.pdf]

Table S3: Enrichment of (A) gene knockout signatures and (B) TFBS for each module.

(A)

Module	# of Enrichments	Knockout Signature	Corrected p-value
1	0		
2	1		
2		SHE4	0.0207028
3	6		
3		AEP2	0.04564
3		DIG1	0.0120993
3		DOT4	0.000754299
3		MED2	9.34194e-005
3		RNR1	1.89422e-005
3		CMD1	0.00389741
4	9		
4		ARG5,ARG6	0.00058713
4		RPL27A	8.32817e-011
4		RPL8A	5.31835e-007
4		SHE4	3.1446e-010
4		CMD1	2.33691e-007
4		YMR014W	4.08593e-014
4		YMR269W	4.69195e-006
4		YOR078W	5.90301e-010
4		CDC42	2.73989e-011
5	0		
6	2		
6		OST3	0.000148772
6		KAR2	7.45556e-005
7	17		
7		ASE1	6.55778e-005
7		CKB2	0.000225305
7		CLB2	4.5188e-009
7		ERG3	0.0441422
7		GCN4	2.62455e-005
7		HDA1	4.7218e-007
7		HPT1	0.00181098
7		IMP2'	0.000575193
7		RTG1	1.0706e-008

7		STE24	1.85193e-005
7		SWI4	1.94842e-009
7		UBR1	3.04524e-005
7		VPS8	1.44793e-007
7		YAP1	0.00728782
7		YER044C	0.0382029
7		YHL029C	3.92602e-009
7		YMR031W-A	1.68972e-009
8	53		
8		ANP1	7.83298e-026
8		ARG80	0.000483452
8		ASE1	3.13982e-010
8		CEM1	1.1635e-006
8		CKB2	9.94202e-029
8		CLB2	6.85266e-034
8		DOT4	0.034653
8		ERG2	1.1114e-009
8		ERG3	1.06824e-049
8		GAS1	2.98768e-014
8		GCN4	6.78244e-010
8		GLN3	2.41239e-015
8		HDA1	2.8158e-043
8		HIR2	5.03605e-006
8		IMP2	0.0108791
8		IMP2'	1.01014e-013
8		ISW2	6.24731e-012
8		JNM1	6.68392e-011
8		NPR2	1.88158e-015
8		PEP12	0.0384115
8		PET117	2.78367e-007
8		PEX12	0.00305798
8		RAD57	1.25475e-015
8		RNR1	1.54884e-005
8		RPL20A	2.60673e-005
8		RRP6	5.4111e-013
8		RTG1	6.23178e-016
8		RTS1	3.45154e-009
8		SBH2	2.04094e-006
8		SGS1	8.84227e-012
8		SIR2	2.97987e-010
8		SIR4	6.73041e-008
8		SPF1	2.30818e-010
8		SST2	5.85599e-014
8		STE24	8.73564e-022

8		SWI4	3.46699e-038
8		SWI5	1.5124e-011
8		UBR1	6.90466e-032
8		VPS8	1.10699e-031
8		YAP1	5.20361e-012
8		YEL008W	1.53204e-048
8		YER044C	3.03825e-046
8		YER083C	9.05711e-032
8		CMD1	5.46727e-017
8		YHL029C	1.68284e-049
8		YHR011W	3.31296e-016
8		YMR010W	2.96556e-012
8		YMR031W-A	8.11328e-051
8		YMR237W	1.05886e-011
8		YOR080W	4.83734e-006
8		AUR1	6.43358e-030
8		ERG11	2.80618e-018
8		PMA1	4.81047e-012
9	7		
9		ANP1	0.00264632
9		ERG2	6.11111e-006
9		SWI4	1.82168e-008
9		YER044C	9.14429e-006
9		YER083C	0.000132062
9		YMR031W-A	1.98126e-007
9		ERG11	0.0202346
10	18		
10		ANP1	0.00173392
10		BUL1	5.3888e-009
10		ERG2	0.00217763
10		ERG3	0.00155711
10		GAS1	0.000259314
10		HDA1	0.00268285
10		MED2	1.24747e-006
10		MSU1	0.0115398
10		RTG1	1.82593e-009
10		RTS1	0.000398795
10		SWI4	0.00324771
10		UBR1	0.000334485
10		VPS8	2.68361e-005
10		YEL008W	0.0342288
10		YER044C	4.24275e-005
10		YER050C	0.00136244

10		YHL029C	0.000322318
10		YMR031W-A	0.000450419
11	3		
11		ERG2	0.00144085
11		CDC42	0.0329592
11		ERG11	0.000133968
12	1		
12		SIR3	0.0021677
13	10		
13		FUS3,KSS1	2.2561e-007
13		SOD1	0.00195799
13		STE11	0.0104319
13		STE12	1.48824e-005
13		STE18	9.75345e-006
13		STE4	0.00047005
13		STE5	0.00443473
13		STE7	3.68709e-006
13		YOR080W	0.0180624
13		CDC42	0.0140429
14	4		
14		DIG1	0.000106811
14		FAR1	0.00034842
14		PPR1	1.90536e-006
14		STE2	0.00118725
15	20		
15		CLA4	0.0212945
15		DIG1,DIG2	2.26045e-009
15		DIG1,DIG2	1.87379e-009
15		FUS3,KSS1	3.5499e-010
15		HOG1	3.71776e-009
15		RAD6	8.67486e-005
15		RAS2	1.91279e-009
15		SOD1	8.19655e-005
15		SST2	1.69074e-009
15		STE11	8.51374e-009
15		STE12	2.87924e-009
15		STE18	8.60216e-010
15		STE24	6.02652e-009
15		STE4	1.87197e-009
15		STE5	2.56628e-010

15		STE7	4.80181e-011
15		SWI6	0.0351961
15		YAL004W	0.00184352
15		YJL107C	0.00128859
15		CDC42	0.00201397
16	0		
17	12		
17		AFG3	0.029035
17		CKB2	0.0010021
17		ERG2	0.000365373
17		HMG1	3.57801e-012
17		SHE4	0.00249603
17		SSN6	0.0010668
17		TOP3	2.24989e-017
17		TUP1	7.09253e-006
17		YHR011W	0.0179532
17		YOR080W	0.000341952
17		ERG11	0.0215873
17		HMG2	2.48643e-007
18	6		
18		CUP5	1.09199e-005
18		ERG2	0.00806549
18		ERG3	8.37229e-005
18		HMG1	3.60978e-006
18		VMA8	3.42774e-005
18		HMG2	2.46519e-005
19	11		
19		AEP2	4.32968e-006
19		DOT4	0.00176373
19		HDF1	0.000834675
19		HOG1	4.98248e-010
19		SWI4	1.03155e-009
19		YER050C	0.0460512
19		YIL037C	1.90456e-007
19		YMR025W	0.0240191
19		YMR029C	1.98643e-006
19		YMR293C	0.000281939
19		KAR2	0.000269038
20	4		
20		MED2	0.000154018

20		TEC1	0.0301387
20		VPS8	0.0308224
20		YHR039C	4.66758e-005
21	2		
21		ERG11	0.0113858
21		PMA1	0.0499661
22	2		
22		RML2	1.26762e-009
22		IDI1	0.0479735
23	0		
24	0		
25	0		
26	33		
26		ADE2	2.62478e-006
26		AEP2	1.35411e-007
26		AFG3	1.71354e-005
26		BIM1	2.85521e-006
26		BUB3	8.11664e-005
26		CKA2	1.49946e-009
26		CKB2	1.80146e-008
26		DOT4	0.000303983
26		GPA2	5.67314e-005
26		ISW1,ISW2	0.00063892
26		KIM4	1.30334e-006
26		MSU1	0.0202058
26		PEP12	2.03048e-006
26		PFD2	0.00819877
26		QCR2	2.3353e-009
26		RML2	0.0038867
26		RNR1	0.0252626
26		RPD3	3.21634e-007
26		RTG1	1.18102e-009
26		RTS1	0.0386478
26		SBP1	2.35665e-009
26		SHE4	2.67608e-009
26		SIR4	6.67666e-010
26		SSN6	3.96719e-008
26		SWI6	0.00142116
26		TUP1	2.4054e-009

26		VAC8	2.43354e-009
26		YEL044W	4.31984e-005
26		YER050C	1.35145e-009
26		CMD1	1.48691e-009
26		YMR293C	8.91152e-005
26		YOR080W	2.50471e-008
26		PMA1	8.50324e-006
27	0		
28	4		
28		BIM1	0.00763734
28		RPD3	0.0156408
28		RPS27B	0.00140615
28		SBP1	0.00154858
29	10		
29		ASE1	0.0360802
29		BIM1	1.86059e-009
29		ERG2	0.00010051
29		GPA2	1.68449e-012
29		MAC1	0.0030108
29		RTS1	1.91767e-009
29		SHE4	1.2768e-009
29		VAC8	2.45084e-006
29		YEL044W	3.08483e-007
29		YOR080W	0.0406953

(**B**)

Module	# of Enrichments	TFBS	Corrected p-value
1	0		
2	0		
3	3		
3		ACE2	2.46E-05
3		FKH1	0.016689
3		SWI5	0.009272
4	0		
5	1		
5		MBP1	0.009584
6	1		
6		INO4	0.043544
7	2		
7		BAS1	0.000132
7		GCN4	0.001992
8	2		
8		GCN4	3.90E-43
8		RTG3	3.44E-09
9	0		
10	1		
10		LEU3	1.07E-09
11	2		
11		FKH1	0.03164
11		SWI6	0.015391
12	0		
13	3		
13		DIG1	0.003063
13		MCM1	0.014358
13		STE12	0.007069
14	0		

15	3		
15		DIG1	5.12E-11
15		STE12	3.22E-10
15		TEC1	3.03E-05
16	0		
17	0		
18	1		
18		HAP1	1.11E-23
19	0		
20	2		
20		BAS1	0.046218
20		ZAP1	9.51E-08
21	0		
22	0		
23	0		
24	0		
25	0		
26	1		
26		MSN4	0.000383
27	0		
28	0		
29	5		
29		HAP1	0.000405
29		HAP2	7.38E-10
29		HAP3	0.00164
29		HAP4	9.64E-11
29		HAP5	2.82E-05
